# Correlates of estimated lifetime cruciate ligament survival inform potential rupture risk reduction strategies: findings from the Exceptional Aging in Rottweilers Study

**DOI:** 10.1038/s41598-023-39288-y

**Published:** 2023-08-25

**Authors:** David J. Waters, Rong Fu, Andres E. Carrillo, Emily C. Chiang, Aimee H. Maras, Seema S. Kengeri, Cheri L. Suckow

**Affiliations:** 1 Center for Exceptional Longevity Studies, Gerald P. Murphy Cancer Foundation, West Lafayette, IN 47906 USA; 2 Department of Veterinary Clinical Sciences and the Center on Aging and the Life Course, Purdue University, West Lafayette, IN 47907 USA; 3 Department of Sociology, Siena College, Loudonville, NY 12211 USA; 4 Department of Exercise Science, School of Health Sciences, Chatham University, Pittsburgh, PA 15232 USA; 5 Franciscan Physician Network, West Lafayette, IN 47906 USA

**Keywords:** Diseases, Risk factors

## Abstract

Cranial cruciate ligament (CCL) rupture is one of the most commonly diagnosed orthopedic conditions of pet dogs, making estimated lifetime cruciate ligament survival an attractive endpoint for studies attempting to define clinical and genetic correlates of rupture risk reduction. Early life experiences contribute significantly to the origins of adult health outcomes, yet our current understanding of modifiable susceptibility factors that drive the high frequency of CCL rupture remains limited. We reasoned that combining lifetime medical history with standardized late-life assessment of lifetime cruciate ligament survival and detailed phenotyping of each dog for selected risk variables would provide a sensitive approach to identify factors that would differentiate between lifelong avoidance versus susceptibility to ligament rupture. Here, we report results of Kaplan–Meier analysis of estimated lifetime cruciate ligament survival and Cox proportional hazards modeling to assess risk variables in a lifetime cohort study of 123 purebred Rottweilers, a breed at high risk for veterinarian-diagnosed CCL rupture. We show that gonad removal during the 24-month developmental period is adversely associated with three measures of susceptibility—increased incidence of CCL rupture, multiplicity (bilateral rupture), and accelerated time to initial CCL failure. Our analysis reveals two other phenotypes—short adult height and the production of offspring (in females)—are associated with significant CCL rupture risk reduction. Together, the results provide clues to an early endocrine influence on lifetime cruciate ligament survival. Further, we identify two distinct clinical syndromes of CCL failure, providing a disease subtyping framework to advance future progress in genetic epidemiology, pathogenesis, and prediction. By conducting an evaluation of estimated lifetime CCL survival in dogs, we show that cruciate ligament survival may be jeopardized by gonad removal during the developmental period. Avoidance of such early environmental adversity may represent an actionable method for the control of canine CCL disease in certain breeds.

## Introduction

Cranial cruciate ligament (CCL) rupture is one of the most commonly diagnosed orthopedic conditions of pet dogs. Once considered to be a consequence of a traumatic insult, it is now believed that the vast majority of mid-substance ruptures of the CCL in dogs are the consequence of a degenerative (non-traumatic) process—a progressive ligament fiber deterioration that can be accelerated by local and systemic factors leading to ligament failure^1,2^. Clinically, the prodromal, pre-rupture phase of this degenerative disease is accompanied by changes (e.g., joint effusion, osteophyte formation) that may be detectable on stifle radiographs prior to onset of pain or stifle lameness^3–5^. The clinical hallmark of CCL rupture diagnosis is excess cranial-caudal stifle joint instability (positive cranial drawer) elicited in a dog with acute onset of stifle pain and lameness. However, in the untreated destabilized stifle, progressive osteophytosis and periarticular fibrosis rather than joint laxity often predominates, which may result in a limited amount of detectable cranial drawer in dogs with chronic CCL rupture^6^. Because the ligament has limited ability to repair in a hostile intraarticular environment, incomplete ruptures in dogs often progress to complete rupture^6^. Similarities and differences between CCL rupture in dogs and anterior cruciate ligament (ACL) rupture in humans have been examined extensively in two recent comparative reviews^1,7^.

Both genetic and environmental factors contribute to the risk of CCL rupture. The importance of genetic susceptibility factors is supported by documented breed differences in the incidence of CCL rupture^8^ and cruciate ligament biomechanical properties^9^. Detailed studies of the genetic etiology of CCL rupture in Labrador Retrievers, a high-risk breed, have shown the risk for CCL rupture to be moderately heritable and highly polygenic, with more than 170 predicted risk loci^10^. Environmental factors may impact ligament vulnerability during critical temporal windows of exposure. A nexus between undergoing gonadectomy, becoming overweight, and increased risk for canine CCL rupture has been postulated^11–16^. A recent report of veterinary hospital-based data on 35 dog breeds suggested that the sensitivity of CCL rupture risk to timing of gonadectomy may be breed-dependent^17^. Unfortunately, many studies fail to report with precision the duration of gonad exposure during the developmental period, instead reporting whether gonads are present or absent at the time of CCL rupture. Other clinical characteristics that may reflect differences in musculoskeletal development and skeletal remodeling, such as adult height, that are distinct from generalized body size, have not been closely evaluated as correlates of CCL rupture in dogs.

To date, retrospective case-comparison studies have provided an incomplete picture of the clinical factors that may promote lifetime cruciate ligament survival. Non-cases (controls) that might later develop CCL rupture are at risk of outcome misclassification because follow-up of non-affected controls does not extend far beyond the date of hospital admittance. Further, not all dogs that develop a CCL rupture go on to develop bilateral rupture; reports estimate only 22–55% will suffer contralateral rupture^4,5^. In many studies of CCL rupture, investigators fail to distinguish between dogs that experience bilateral versus unilateral CCL rupture^8,11,15–22^. As a result, explorations of risk factors have relied mainly upon comparison of controls versus dogs with any rupture or comparison of dogs with unilateral versus bilateral rupture. Taken together, these methodological limitations have not allowed investigators to conduct a detailed comparison of dogs with *lowest susceptibility* to CCL rupture (i.e., lifelong avoidance of CCL rupture) versus dogs with *highest susceptibility* (i.e., bilateral CCL rupture).

Recently, findings from the largest epidemiological study of canine CCL rupture were reported using veterinary insurance claims in a cohort of over 600,000 dogs living in Sweden^8^. This important study showed a markedly higher incidence of CCL rupture diagnosis among breeds of large body size, a female preponderance for CCL rupture, and earlier age at ligament rupture among breeds at highest risk. However, patient-level data on duration of gonad exposure, bilateral vs. unilateral rupture, adult height, or body condition were unavailable in this study and in another epidemiological study^23^, elevating the need for a complementary study that could evaluate these potential correlates of lifetime cruciate ligament survival.

The Exceptional Aging in Rottweilers Study (EARS) seeks to better understand the biology of cellular aging and disease resistance by discovering genetic and environmental correlates of morbidity compression and longevity through the detailed study of the oldest-living Rottweilers in North America. Its ambidirectional cohort study design—consisting of both retrospective and prospective acquisition of extensive data from dogs reaching exceptional longevity—includes a subset of dogs that underwent a standardized orthopedic examination by a veterinarian experienced in the physical diagnosis of CCL rupture conducted after 13 years of age, representing an age four years greater than the median lifespan of the breed^24,25^. Using this methodological approach—establishing the presence or absence of veterinarian-diagnosed CCL rupture based on detailed review of medical history, combined with assessment of abnormal laxity or periarticular fibrosis during a standardized near-end-of-life stifle examination—enabled lifetime cruciate ligament survival to be estimated in a cohort of 123 dogs from the EARS study.

We reasoned that by establishing an estimate of lifetime cruciate ligament survival from birth to 13 years in this cohort of Rottweiler dogs, one could test for associations between selected risk variables and CCL outcome unencumbered by censoring, because no dogs died or were lost to follow-up during the observation period. This would strengthen the ability to detect the existence of early versus late onset syndromes, and to identify factors that may accelerate development of CCL rupture. Because early, prodromal synovitis and inflammatory changes are believed over time to progress toward fiber disruption, which may or may not culminate in complete CCL rupture in untreated stifles^6^, assessment of CCL status near-end-of-life would allow early events in affected stifles to progress to more clinically discernible CCL outcomes, which should aid in minimizing outcome misclassification. Moreover, detection of CCL rupture at the time of standardized examination in dogs having no previous medical history of CCL rupture diagnosis would decrease the false negative rate inherent in clinical detection studies, providing an enriched likelihood of demonstrating significant associations between risk variables and CCL outcome.

Here, we report the results of the first analysis of the clinical correlates of estimated lifetime cruciate ligament survival in Rottweilers using data from the EARS study. We provide evidence of a gonad exposure window during development that is associated with risk of bilateral CCL rupture, but not unilateral rupture. Moreover, we report two other phenotypes—short adult height and the production of offspring (in females)—are associated with significant CCL rupture risk reduction. Finally, our analysis points to two distinct clinical syndromes of CCL failure in Rottweilers, suggesting a subtyping framework that may provide new opportunities for further genetic discovery and fresh momentum for the pursuit of personalized risk reduction strategies.

## Results

### General description of study cohort

The study cohort consisted of 123 purebred Rottweilers that received standardized assessment of CCL status after they reached 13 years of age. The proportion of dogs with estimated lifetime resistance to CCL rupture—those dogs with lifelong avoidance of veterinarian-diagnosed CCL rupture—was 81 of 123 (66%). Forty-two dogs sustained a total of 60 CCL ruptures. Twenty-four dogs had unilateral CCL rupture, whereas 18 dogs had bilateral rupture. Among dogs with bilateral rupture, median (range) interval from first to second rupture was 1.3 (0.1–6.7) years. Thirty-eight CCL ruptures were diagnosed at the time of open surgical treatment. Twenty-two ruptures in non-surgically treated stifles were confirmed at the near-end-of-life standardized orthopedic examination. Six of these dogs had ruptures that were occult, with no medical history of a stifle event, no diagnosis of CCL rupture until revealed by the near-end-of-life assessment (Supplementary Figure S1).

Females outnumbered males in the study cohort (76F, 47M), but females were not more likely to suffer CCL rupture (*p* = 0.32) (Supplementary Table S1). Median (IQR) duration of gonad exposure in the cohort was 5.0 (2.0–7.9) years; 33 (27%) of 123 dogs experienced gonad removal during the first 24 months of life (EED_24m_). Reason for gonad removal attributed to conformational abnormality/orthopedic conditions was limited to seven (6%) of the 109 dogs that underwent gonadectomy. No dogs underwent gonad removal with the intent of treating an orthopedic condition.

Estimated CCL rupture incidence rates are shown in Table 1. Incidence rate for males and females combined was 314 cases per 10,000 dog-years at risk. Incidence rate in dogs that underwent gonad removal during the first 24 months of life was more than four times higher than in dogs that retained their gonads longer than 24 months. Female : male risk ratio was 1.01 after excluding dogs that underwent early gonad removal (Table 1).

**Table 1:** Comparison of cranial cruciate ligament (CCL) rupture incidence rates in Rottweilers reported in an insured Swedish dog population (ref 8) versus incidence rates in a cohort of 123 dogs from the Exceptional Aging in Rottweilers Study (EARS).

	Affected Dogs (Cases)	DYAR	Incidence (95% CI) (Cases/10,000 DYAR)	F:M Ratio
Insured Swedish Dog Population^8^				
Females & males	–	–	127 (112–143)*	1.13:1**
Exceptional Aging in Rottweilers Study				
Females and males, 0–13 years	42	1326	317 (228–428)	1.43:1
Females only	28	772	362 (241–524)	–
Males only	14	554	253 (138–424)	–
Females and males, EED_24months_†	21	262	802 (496–1225)	–
Females and males, excluding EED_24months_††	21	1064	197 (122–302)	1.01:1
Females only	9	454	198 (91–376)	–
Males only	12	610	197 (102–344)	–
Females and males, 2–10 years, excluding EED_24months_†††	10	676	148 (71–272)	–

*DYAR* dog years at risk, *F:M ratio* female: male ratio, *95% CI* 95% confidence interval, *EED*_*24months*_ early endocrine disruption (EED) caused by gonad removal during the developmental period, i.e., the first 24 months_(24 months)_ of life.

*Based upon published data on Swedish Rottweilers (ref 8).

**Based upon published data on all breeds (ref 8).

^†^Relative risk for CCL rupture in females and males with early endocrine disruption (EED) during first 24 months of life compared to non-EED females and males is 802/197 = 4.1.

^††^In this subgroup of dogs in the EARS cohort that excludes dogs that underwent early gonad removal, female : male ratio of incidence rates is expressed as 1.01:1 (198/197) indicating that, when data were analyzed free of confounding by early gonad removal, the risk for CCL rupture in females versus males in the EARS cohort approaches unity.

^†††^It is notable that the CCL rupture incidence rate in this subgroup of dogs in the EARS cohort most closely approximates the incidence rate reported in the Swedish Rottweiler population (depicted above) as its selection criteria attempts to mimic the age restriction of some of the insured dogs in the Swedish cohort and also excludes dogs in the EARS cohort that underwent early gonad removal because this is uncommonly performed in pet dogs in Sweden.

A descriptive summary of characteristics of the 123 dogs in this study cohort is presented in Table 2. Supplementary Table S2 summarizes characteristics of the three CCL outcome groups: Resistant (n = 81 dogs); Unilateral Rupture (n = 24 dogs); Bilateral Rupture (n = 18 dogs).

**Table 2:** Summary of characteristics of 123 dogs in the Exceptional Aging in Rottweilers Study.

Variable		Total (n = 123)
Residence	Number of U.S. states	37 states and Canada
	Number of households	119
Sex, #Females, #Males		76F, 47M
Age at Death, median (range), in years		14.1 (13.1–16.5)
Age at CCL Status Ascertainment, median (range), in years		13.5 (13.0–15.9)
Date of CCL Status Ascertainment		March 2010–Feb 2020
Lifetime CCL Outcome	Lifelong ligament survival, n (%)	81 (65)
	Unilateral rupture, n (%)	24 (20)
	Bilateral rupture, n (%)	18 (15)
Age at First CCL Rupture	Overall (n = 38), median (range), in years	6.0 (2.0–13.6)
	Unilateral (n = 20), median (range), in years	9.9 (3.8–13.6)
	Bilateral (n = 18), median (range), in years	3.9 (2.0–8.7)
Surgical Repair of CCL Rupture (CCLR)	Surgically treated dogs, n (% dogs with CCLR)	24 (57)
	Number of operations	38 operations in 24 dogs
	TPLO technique, n (% operations)	20 (53%)
	Injury to surgery interval, median (IQR), in weeks	2.0 (0.9–4.0)
Age at Gonad Removal	Females (N = 76), median (IQR), in years	4.4 (1.1, 6.8)
	Females ≤ 24 months (EED_24m_), n (%)	23 (30)
	Males (N = 47), median (IQR), in years	6.8 (2.5, 13.5)
	Males ≤ 24 months (EED_24m_), n (%)	10 (21)
Reason for Gonad Removal	Abn Ortho/Conform, n (%)	7 (6)
	Other reasons, n (%)	103 (94)
Lifetime Gonad Retention, #Females, #Males		0F, 14 M
Adult Height	Females (N = 76), median (range), in cm	61.0 (54.6–67.3)
	Males (N = 47), median (range), in cm	63.5 (61.0–68.6)
Reproductive Status in 53 Females with > 24 m Ovary Exposure	Parous, n (% of reproductively mature females)	21 (40)
Body Condition	Precedent overweight, n (%)	20 (16)
Cause of Death/Euthanasia	Sudden death, n (%)	14 (11.4)
	Frailty, n (%)	19 (15.4)
	Cancer, n (%)	30 (24.4)
	Other, n (%)	40 (32.5)
	Insufficient information, n (%)	12 (9.8)
	DJD, n (%)	8 (7.2)

*Abn Ortho/Conform* abnormal conformation/orthopedic abnormality, *CCL* cranial cruciate ligament, *DJD* = degenerative joint disease, *EED*_*24m*_ early endocrine disruption (EED) caused by gonad removal during the developmental period, i.e., the first 24 months_(24 m)_ of life, *F,M* female, male, *IQR* interquartile range, *Parous* females producing offspring, *TPLO* tibial plateau leveling osteotomy, # number.

### Gonad exposure window analysis reveals a significant association between increased risk of CCL rupture and early endocrine disruption

To determine the relationship between age at gonad removal and risk of CCL rupture, we conducted a gonad exposure window analysis. This enabled a comparative evaluation of CCL rupture risk associated with gonad removal during three successive time intervals in each dog’s 24-month developmental period (gonad removal at age ≤ 6 months, 6.01–12 months, 12.01–24 months). Compared to dogs that retained gonads for more than 24 months, gonad removal during each of the developmental windows was associated with a significant increase in risk for CCL rupture (Table 3). Dogs with the shortest gonad exposure (≤ 6 months) had the highest risk for any CCL rupture [unadjusted HR (95% CI) = 10.34 (4.59–23.31); *p* < 0.001]. After adjustment for sex, adult height, and body condition, the strong association between early gonad removal and risk for CCL rupture remained significant in each of the three gonad exposure windows [for ≤ 6 months, HR_adjusted_ (95% CI) = 8.33 (3.49–19.88); *p* < 0.001] (Table 3). The decision to remove or retain gonads between the ages of 2–4 years, 4–6 years, or 6–8 years had no significant association with CCL rupture risk (Supplementary Table S3). Together, these findings reinforced our rationale for using 24 months as the cut point for gonad exposure in our main analyses.

**Table 3:** Association between age at gonad removal and risk for any cranial cruciate ligament (CCL) rupture: An analysis of three distinct windows of gonad exposure (≤ 6 months, 6.01–12 months, 12.01–24 months) during the developmental period.

	Gonad exposure	Hazard ratio	95% CI	*p*-value
	> 24 months (n = 90)	1.0 (ref)	–	–
Windows During Developmental Period	12.01–24 months (n = 12)	3.43	1.45–8.10	0.005
		3.40*	1.42–8.15	0.006
	6.01–12 months (n = 10)	3.17	1.19–8.44	0.02
		2.95*	1.08–8.09	0.04
	< 6 months (n = 11)	10.34	4.59–23.31	< 0.001
		8.33*	3.49–19.88	< 0.001

For each developmental window, hazard ratio (HR) and 95% confidence interval (95% CI) for CCL rupture were generated using Cox proportional hazards modeling using dogs with > 24 months gonad exposure as reference (ref) group (HR = 1.0). n = 123.

*Represents the adjusted hazard ratio (HR) including sex, adult height, and body condition in multivariate analysis.

### Likelihood of tall adult height, a risk factor for CCL rupture, is increased in dogs with early gonad removal

First, we analyzed the relationship between adult height and CCL rupture using adult height as a continuous variable. In 76 females, risk for CCL rupture increased almost twofold for every 2.5 cm increase in height [HR (95% CI) = 1.91 (1.28–2.86); *p* = 0.002)]. In 47 males, risk for CCL rupture associated with taller height was even larger [HR (95% CI) = 3.25 (1.45–7.24); *p* = 0.004]. Cox proportional hazards analysis using sex-specific cut points for short versus tall (tall females ≥ 61.0 cm, tall males ≥ 64.8 cm) and sex-specific height tertiles yielded the same conclusion: short adult height was associated with significant CCL rupture risk reduction (Supplementary Table S4).

Because early gonad removal can delay closure of epiphyseal growth plates, thereby potentially promoting additional long bone growth, we determined whether gonad removal during the sensitive developmental period increased the likelihood of reaching tall adult height. Dogs that experienced gonad removal during the first 6 months of life were on average 3.2 cm taller than dogs that retained their gonads for > 24 months (*p* = 0.03) (Supplementary Table S5). Using sex-specific height cut points, dogs that had EED_24m_ were 2.4X more likely to be tall compared to dogs that retained their gonads during the developmental period [OR (95% CI) = 2.40 (1.08–5.56; *p* = 0.03]. These observations raise the prospect that the likelihood of achieving tall adult height might be modifiable by avoiding early gonad removal.

### Estimated lifetime cruciate ligament survival is associated with adult height and duration of gonad exposure

**Figure 1:**
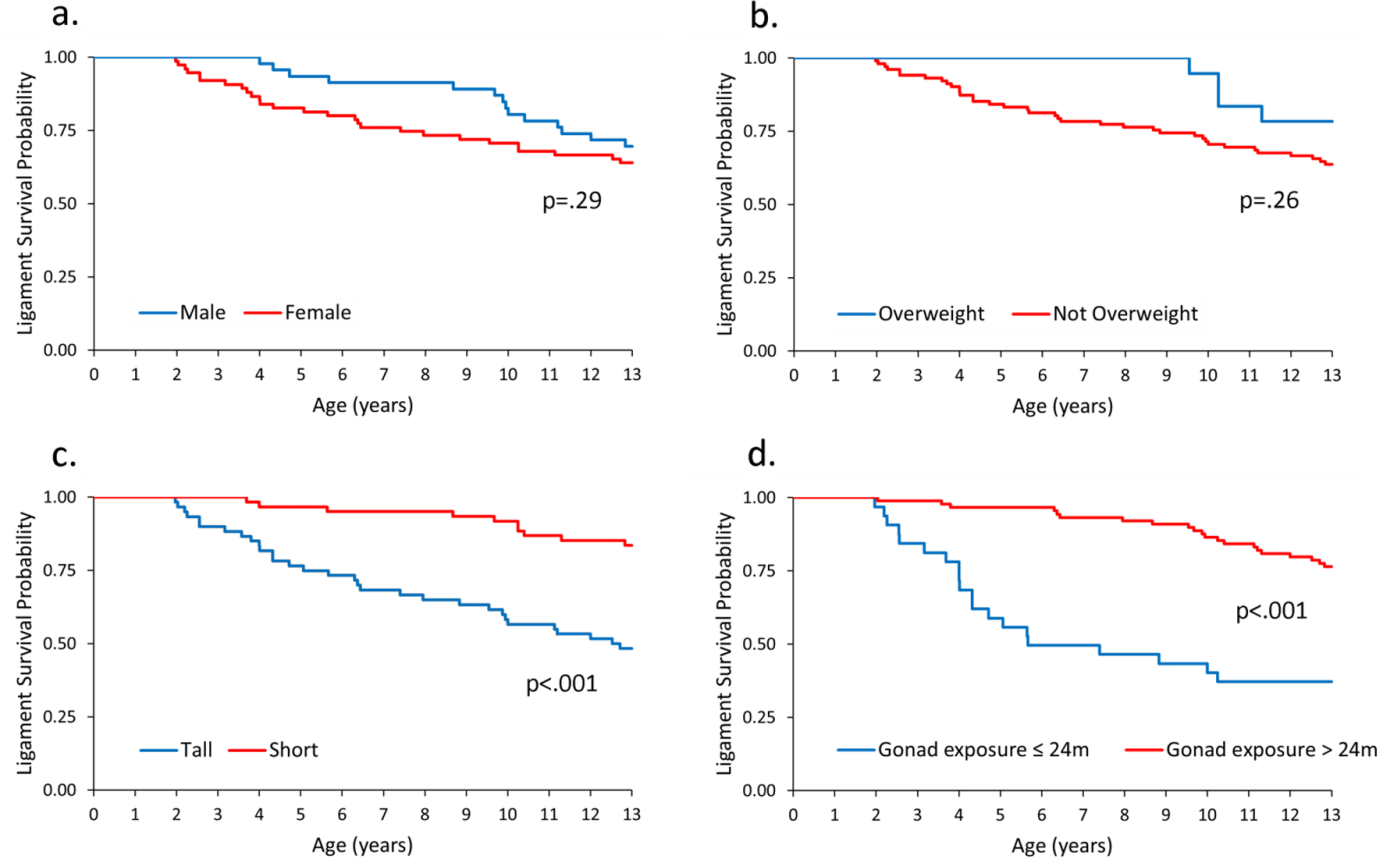
Kaplan–Meier curves for lifetime cruciate ligament survival in the Exceptional Aging in Rottweilers Study (EARS) cohort (n = 123). Four panels show cruciate ligament survival stratified by (**a**) sex, (**b**) body condition, (**c**) adult height, and (**d**) gonad exposure. In this survival analysis, time to event is time to first rupture, i.e., loss of any CCL survival. Overweight versus not overweight refers to body condition prior to the time of first rupture, i.e., presence or absence of precedent overweight. Tall versus short height reflects sex-specific cut points of adult height (TALL females > 61.0 cm; TALL males > 64.8 cm). Gonad exposure of ≤ 24 months versus > 24 months represents dogs that experienced gonad removal versus gonad retention during the developmental period, i.e., first 24 months of life. *P*-values calculated using log-rank test.

Kaplan–Meier survival analysis was used to determine if there were differences in estimated lifetime cruciate ligament survival between groups stratified by sex, body condition (overweight versus not overweight), adult height (short vs. tall), and gonad exposure (≤ 24 months vs. > 24 months). Lifetime cruciate ligament survival was not different between males and females (*p* = 0.29) or between dogs that had overweight versus not overweight body condition (*p* = 0.26) (Fig. 1, panel A and B). In contrast, dogs with short adult height had a lifetime cruciate ligament survival advantage over tall dogs (*p* < 0.001) (Fig. 1, panel C). Similarly, dogs that did not experience EED_24m_ had a significant lifetime cruciate ligament survival advantage over dogs with early endocrine disruption (*p* < 0.001) (Fig. 1, panel D).

**Figure 2:**
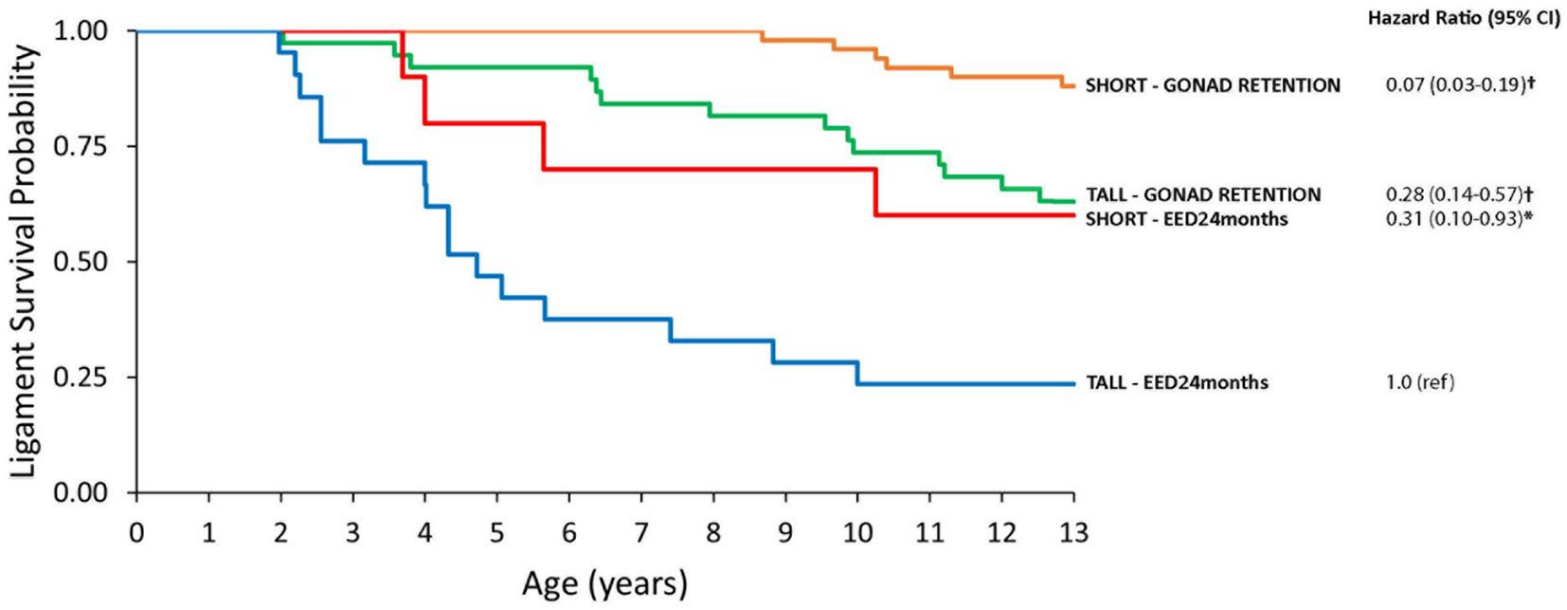
Kaplan–Meier curves for lifetime cruciate ligament survival in the Exceptional Aging in Rottweilers Study (EARS) cohort (n = 123). Four curves show cruciate ligament survival stratified across four combinations of two risk variables (adult height, gonad exposure): SHORT–GONAD RETENTION (n = 51); SHORT–EED24months (n = 11); TALL–GONAD RETENTION (n = 39); TALL–EED24months (n = 22). EED24months = early endocrine disruption (EED) caused by gonad removal during the developmental period, i.e., the first 24 months of life. Hazard ratio (HR) and 95% confidence interval (95% CI) generated using Cox proportional hazard modelling using TALL-EED24months as reference (ref) group (HR = 1.0). In this survival analysis, time to event is time to first rupture, i.e., loss of any CCL survival. **†**differs from reference group, *p* < .001. *differs from reference group, *p* = .02.

To evaluate further the relationship between adult height, gonad exposure, and lifetime cruciate ligament survival, Kaplan–Meier curves were constructed to depict differences in ligament survival across four combinations of two dichotomous risk variables: TALL and EED_24m_ (n = 22 dogs); TALL and GONAD RETENTION (n = 39 dogs); SHORT and EED_24m_ (n = 11 dogs); SHORT and GONAD RETENTION (n = 51 dogs) (Fig. 2). Compared to the group of dogs at highest risk for CCL rupture (i.e., TALL and EED_24m_), the SHORT and GONAD RETENTION group had the highest lifetime cruciate ligament survival, exhibiting a 93% lower risk of CCL rupture [HR (95% CI) = 0.07 (0.03–0.19); *p* < 0.001]. Dogs in the intermediate CCL rupture risk categories also experienced a significantly lower lifetime risk of CCL rupture. Kaplan–Meier analysis using 6 months or 12 months as cut points for gonad exposure yielded similar results. After demonstrating these strong correlates of estimated lifetime cruciate ligament survival, we next asked whether we could pinpoint correlates of bilateral CCL failure.

### Bilateral CCL rupture risk reduction is associated with short adult height and avoidance of early endocrine disruption

We reasoned that a direct comparison between dogs with the highest resistance to CCL rupture (lifelong avoidance of veterinarian-diagnosed CCL rupture) and dogs with the highest susceptibility to CCL rupture (bilateral rupture) would maximize signal strength related to exposure—outcome. To pursue this line of thinking, a Cox proportional hazards model of bilateral CCL rupture was used to estimate risk reduction associated with sex, height, gonad exposure, reason for gonad removal, and body condition (Table 4). Compared to EED_24m_, longer gonad exposure was associated with a 95% reduction in risk of bilateral CCL rupture after adjustment for these other risk variables [HR_adjusted_ (95% CI) = 0.05 (0.02–0.20); *p* < 0.001]. The gonad exposure window associated with the strongest risk for bilateral CCL rupture was the first 6 months of life (Supplementary Table S6).

**Table 4:** Unadjusted and adjusted hazard ratios for bilateral cranial cruciate ligament (CCL) rupture generated from Cox proportional hazards model of dogs with bilateral CCL rupture (n = 18) and dogs resistant to CCL rupture (n = 81).

Variable	Unadjusted HR (95% CI)	*p*-value	Adjusted HR (95% CI)	*p*-value
Sex				
Male	1.0 (ref)			
Female	2.36 (0.78–7.18)	0.13	2.89 (0.92–9.12)	0.07
Gonad Exposure				
≤ 24 months	1.0 (ref)			
> 24 months	0.05 (0.02–0.19)	< 0.001	0.05 (0.02–0.20)	< 0.001
Adult Height				
Tall	1.0 (ref)			
Short	0.19 (0.06–0.56)	0.003	0.30 (0.10–0.94)	0.04
Body Condition*				
Precedent overweight	1.0 (ref)			
Not overweight	––			
Reason for Gonad Removal				
Conformation defect/Orthopedic condition	0.77 (0.10–5.77)	0.80	0.33 (0.04–2.75)	0.30
Other	1.0 (ref)			
Age at CCL Status Ascertainment				
13–13.5 years	1.0 (ref)			
> 13.5. years	0.57 (0.22–1.47)	0.24	0.40 (0.15–1.09)	0.08

In this survival analysis, time to event is time to second CCL rupture. For each risk variable, adjusted HR includes the other five variables in a multivariate analysis.

*Body condition drops out of model because none of 18 Rottweilers with bilateral CCL rupture were categorized as precedent overweight prior to the time of first rupture or became overweight during the time prior to the second rupture. Gonad exposure categories: ≤ 24 months = early endocrine disruption; > 24 months = avoidance of gonad removal during the first 24 months of life. Tall females ≥ 61.0 cm; Tall males ≥ 64.8 cm. HR (95% CI) = Hazard Ratio and 95% confidence interval. ref = reference group.

Short adult height was also significantly linked to bilateral CCL rupture risk reduction. Compared to tall dogs, short dogs had a 70% reduction in the multivariate model [HR_adjusted_ (95% CI) = 0.30 (0.10–0.94); *p* = 0.04] (Table 4). In contrast, overweight body condition was not associated with risk of bilateral rupture; none of the 18 dogs that experienced bilateral CCL rupture were overweight prior to the time of first rupture or became overweight prior to the second rupture. Taken together, the results suggested that two phenotypes—short adult height and gonad retention during the first 24 months of life—are strongly associated with lifelong avoidance of bilateral CCL rupture.

### Sensitivity analysis using simulated misclassification of CCL outcome supports the robustness of associations between gonad exposure, adult height, and bilateral CCL risk

Because misclassification of dogs assigned to the three categories of CCL outcome—Resistant, Unilateral Rupture, or Bilateral Rupture—may lead to spurious associations between risk variables and CCL outcome, we conducted random reassignment sensitivity analysis to test the robustness of our main findings. False negative results—dogs scored as Resistant that actually had Unilateral Rupture, dogs scored as Unilateral Rupture that actually had Bilateral Rupture—are of particular concern to investigators. So we re-analyzed the associations between short adult height and avoidance of early endocrine disruption with bilateral CCL rupture risk reduction. After employing category-specific, random outcome reassignment to simulate a 20% false negative rate, 5% false positive rate in our reported CCL outcomes, logistic regression showed that effect size and statistical significance of the main findings were not markedly attenuated by simulated outcome misclassification. Before and after random reassignment of CCL outcome, dogs that avoided gonad removal during the 24-month developmental period experienced at least a 95% reduction in risk of bilateral CCL rupture [OR (95% CI) = 0.05 (0.01–0.20); *p* < 0.001 (Model 2) compared to OR (95% CI) = 0.03 (0.01–0.15); *p* < 0.001 using original data set] (Supplementary Figure S2, panel B). Similarly, dogs with short adult height had a comparable reduction in risk of bilateral CCL rupture before and after simulated misclassification [OR (95% CI) = 0.23 (0.06–0.88); *p* = 0.03 (Model 2) compared to OR (95% CI) = 0.21 (0.05–0.92); *p* = 0.04 using original data set]. Significant bilateral CCL rupture risk reduction associated with short adult height and gonad retention persisted even after more severe outcome reassignment simulating 40% false negative rate, 5% false positive rate (Supplementary Figure S2, panel B, Model 4).

### Increased risk for bilateral CCL rupture associated with early gonad removal is mediated by early age at first rupture, not by height or body condition

If bilateral CCL rupture represents a syndrome of high susceptibility, then bilateral rupture could be the consequence of an accelerated failure process. Consistent with this notion, we found that dogs with bilateral CCL rupture had earlier age at first rupture than dogs with unilateral rupture. Overall, median (IQR) age at first rupture was 6.0 (3.8–10.1) years. In dogs with bilateral CCL rupture, median (IQR) age at first rupture was 3.8 (2.5–5.2) years compared to 9.9 (6.8–11.8) years for dogs with unilateral rupture (*p* < 0.001).

Next, we used logistic regression to identify factors associated with early vs late (< 6 years vs. > 6 years) age at first CCL rupture (Supplementary Table S7 Endocrine disruption during the first 24 months of life was associated with a 14-fold higher likelihood of first CCL rupture prior to 6 years [OR_adjusted_ (95% CI) = 13.99 (2.67–73.3); *p* = 0.002]. Avoidance of early endocrine disruption was associated with an estimated 5.5-year postponement of first CCL rupture (Supplementary Table S8). Tall height, a risk factor for bilateral rupture in this cohort, was not associated with earlier age at first rupture (*p* = 0.34). Among dogs with bilateral rupture, there was a trend for earlier age at first rupture to be associated with shortened interval to contralateral ligament failure (Spearman r = 0.431; *p* = 0.10) (Supplementary Figure S3), consistent with the notion that bilateral CCL rupture is the product of an accelerated failure process. Among dogs with CCL rupture, later age at first rupture was associated with strong reduction in risk for bilateral rupture, even after adjustment for developmental gonad exposure (Supplementary Table S9). In this cohort, none of 15 dogs in which first CCL rupture occurred after 9 years of age experienced bilateral rupture.

**Figure 3:**
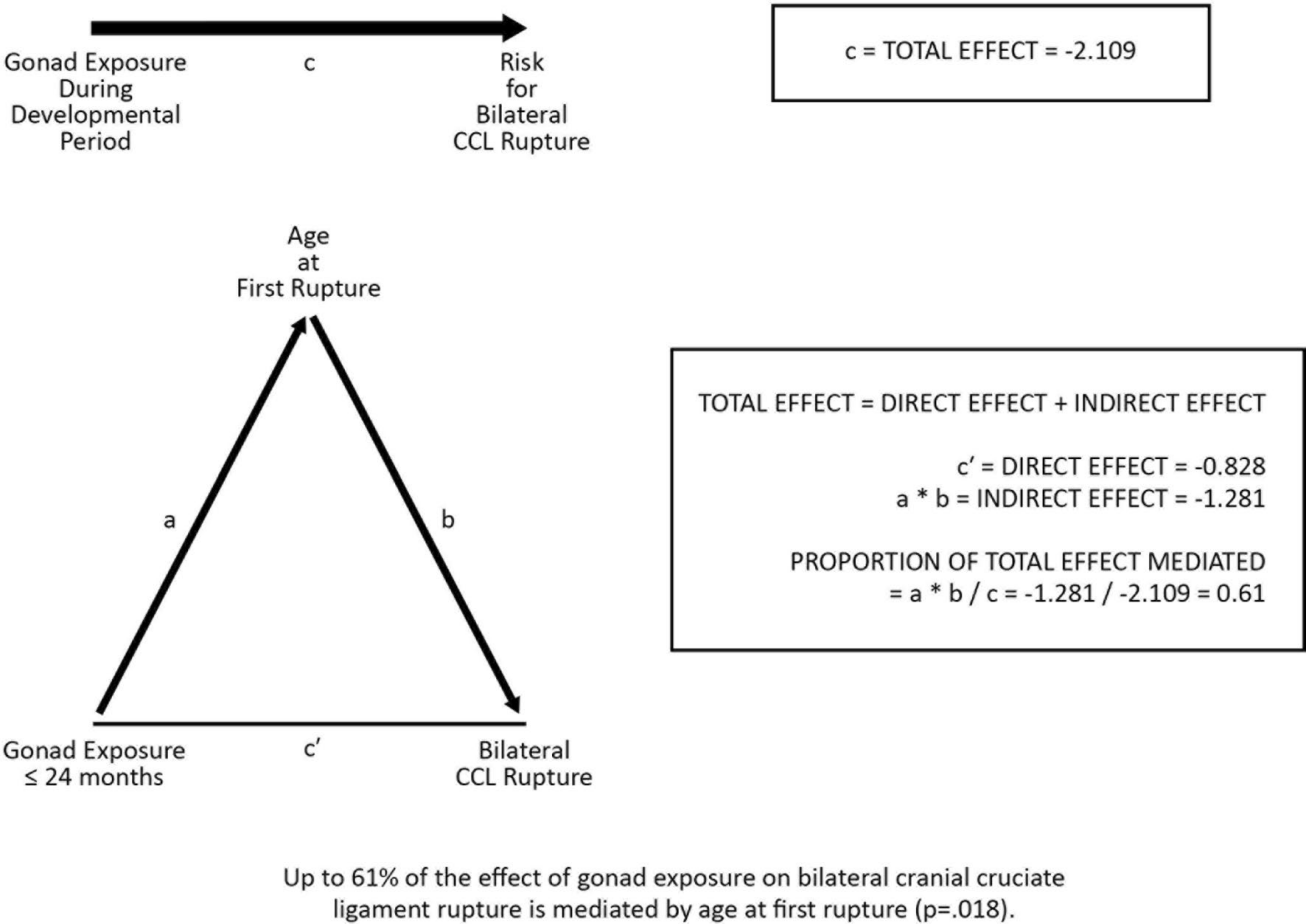
Analysis of early age at first rupture in 38 dogs as a mediator of the effect of gonad removal during the developmental period (gonad exposure ≤ 24 months) on risk of bilateral cranial cruciate ligament (CCL) rupture. Horizontal arrow at top of figure represents total effect (c). Triangle shows direct effect (c´) and indirect effect (a*b). Results are presented with age at first rupture treated as a continuous variable; bilateral CCL rupture (yes/no) is the binary outcome variable.

We then performed mediation analysis using generalized structural equation modeling to estimate the extent to which early age at first rupture mediated the impact of early endocrine disruption on risk of bilateral CCL rupture. We found that up to 61% of the early endocrine disruption impact on bilateral CCL rupture was mediated indirectly through earlier age at first rupture (*p* = 0.018) (Fig. 3). Neither tall height nor being overweight were significant mediators of this effect.

### Unilateral CCL rupture risk reduction is associated with short adult height but not avoidance of early endocrine disruption

Next, we evaluated factors that could contribute to unilateral CCL rupture risk reduction. Cox proportional hazards model for unilateral rupture showed that avoidance of EED_24m_ was not associated with significant risk reduction for unilateral rupture [HR_adjusted_ (95% CI) = 0.66 (0.26–1.68); *p* = 0.38] (Table 5). However, short adult height was associated with a 78% risk reduction of unilateral rupture compared to tall dogs [HR_adjusted_ (95% CI) = 0.22 (0.08–0.57); *p* = 0.002]. Avoidance of overweight body condition prior to unilateral CCL rupture was not associated with significant risk reduction [HR_adjusted_ (95% CI) = 0.76 (0.26–2.21); *p* = 0.62]. Thus, short adult height carries a similar magnitude of risk reduction for unilateral CCL rupture as it does for bilateral CCL rupture, 78% versus 70% reduction in risk, respectively. The significant association between short adult height and unilateral rupture risk reduction was attenuated by simulated outcome misclassification [OR (95% CI = 0.49 (0.20–1.18); *p* = 0.11] to mimic a 20% false negative rate, 5% false positive rate and also after 40% false negative rate, 5% false positive rate (See Supplementary Figure S2 panel B, Model 2 and Model 4). Notably, our work suggests risk for unilateral and bilateral CCL rupture are differentially affected by gonad removal, a finding that persisted during sensitivity analysis.

**Table 5:** Unadjusted and adjusted hazard ratios for unilateral cranial cruciate ligament (CCL) rupture generated from Cox proportional hazards model of dogs with unilateral CCL rupture (n = 24) and dogs resistant to CCL rupture (n = 81). In this survival analysis, time to event is time to first CCL rupture.

Variable	Unadjusted HR (95% CI)	*p*-value	Adjusted HR (95% CI)	*p*-value
Sex				
Male	1.0 (ref)			
Female	1.00 (0.44–2.25)	0.99	0.86 (0.37–2.00)	0.72
Gonad Exposure				
≤ 24 months	1.0 (ref)			
> 24 months	0.56 (0.22–1.41)	0.22	0.66 (0.26–1.68)	0.38
Adult Height				
Tall	1.0 (ref)			
Short	0.22 (0.09–0.56)	0.001	0.22 (0.08–0.57)	0.002
Body Condition				
Precedent overweight	1.0 (ref)			
Not overweight	0.93 (0.35–2.50)	0.89	0.76 (0.26–2.21)	0.62
Reason for Gonad Removal*				
Conformation defect /Orthopedic condition	––			
Other	1.0 (ref)			
Age at CCL Status Ascertainment				
13—13.5 years	1.0 (ref)			
> 13.5 years	0.79 (0.35–1.76)	0.56	0.98 (0.43–2.27)	0.97

For each risk variable, adjusted HR includes the other five variables in a multivariate analysis. *Reason for gonad removal drops out of model because none of 24 Rottweilers with unilateral CCL rupture were categorized as conformation defect/orthopedic condition prior to gonad removal. Gonad exposure categories: ≤ 24 months = early endocrine disruption; > 24 months = avoidance of gonad removal during the first 24 months of life. Tall females ≥ 61.0 cm; Tall males ≥ 64.8 cm. Body Condition categories: (1) overweight prior to CCL rupture (i.e., precedent overweight); or (2) not overweight prior to CCL rupture (i.e., not overweight). HR (95% CI) = Hazard Ratio and 95% confidence interval. ref = reference group.

### Production of offspring (parity) is associated with CCL rupture risk reduction in tall females that do not undergo gonad removal during the 24-month developmental period

Because the strong association between gonad removal and CCL rupture suggested that cruciate ligament disease may be sensitive to alterations in sex hormones, we investigated whether the production of offspring (parity) influenced veterinarian-diagnosed CCL rupture risk in the subgroup of tall females that retained their ovaries and reached reproductive maturity. Cranial cruciate ligament rupture occurred in 8 of 15 (53%) non-parous females. In contrast, only 2 of 11 (18%) parous females developed CCL rupture; in both of these cases, rupture occurred prior to first whelping. Compared to non-parous females, parous females had a 93% reduction in the risk of CCL rupture [HR_adjusted_ (95% CI) = 0.07 (0.01–0.81); *p* = 0.03] (Supplementary Table S10). This finding provides further supporting evidence that CCL disease initiation and/or progression to CCL rupture may be sex hormone-sensitive.

### Two distinct CCL failure syndromes are revealed by analysis of estimated lifetime cruciate ligament survival in Rottweilers

**Figure 4:**
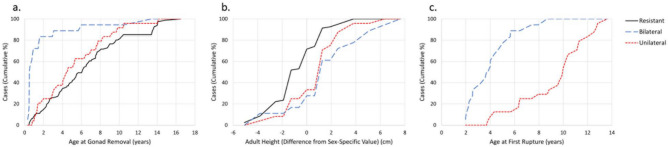
Cumulative Frequency Distribution for dogs with three categories of lifetime cranial cruciate ligament (CCL) outcome (Resistant, Bilateral, Unilateral) based upon (**a**) age at gonad removal, (**b**) adult height, and (**c**) age at first rupture. Resistant = lifelong cruciate ligament survival; Bilateral = bilateral CCL rupture; Unilateral = unilateral CCL rupture. Panel A: the frequency of earlier age at gonad removal in the bilateral CCL rupture group clearly exceeds that of dogs in the unilateral CCL rupture group and the CCL rupture-resistant group. Panel B: in dogs in the bilateral and unilateral CCL rupture groups, a similar frequency distribution of adult height values is observed. The frequency of shorter adult height (negative values representing height less than sex-specific breed standard values) in CCL rupture-resistant dogs exceeds that of dogs with bilateral or unilateral CCL rupture. Panel C: the frequency of earlier age at first rupture in the bilateral CCL rupture group clearly exceeds that of dogs in the unilateral CCL rupture group. Overall, the analysis suggests that two risk variables—age at gonad removal (i.e., gonad exposure) and age at first rupture (a proxy for accelerated ligament failure)—may be useful in the prediction of cruciate ligament rupture susceptibility (i.e., bilateral versus unilateral rupture).

To determine if susceptibility to cruciate ligament failure is accompanied by detectable differences in clinical phenotypes, we used cumulative frequency distribution function to compare the degree to which dogs with bilateral versus unilateral CCL rupture differed in terms of other clinically measurable characteristics (Fig. 4). On the basis of age at rupture, age at gonad removal, and multiplicity (bilateral vs. unilateral rupture), affected dogs could be categorized into two distinct CCL failure syndromes: (1) early, gonad-sensitive bilateral rupture; and (2) later onset, gonad-insensitive unilateral rupture. In bilateral cases, more than 85% of first ruptures occurred at less than 6 years, an age at which 85% of dogs destined to develop unilateral rupture had not yet been affected (Fig. 4, panel C). More than 80% of bilateral cases had EED_24m_, whereas 80% of unilateral cases had gonad exposure that exceeded 24 months (Fig. 4, panel A). Of the 34 dogs with CCL rupture that had either of the two discriminating exposure dyads (i.e., EED_24m_ and EARLY RUPTURE; GONAD RETENTION and LATE RUPTURE), the diagnosis of bilateral versus unilateral rupture could be accurately predicted in 91% (95% CI = 77–97%) of cases. Neither adult height (Fig. 4, panel B) nor sex was a distinguishing feature of the two syndromes. Altogether, our data in Rottweilers suggest the existence of two distinct CCL failure syndromes, including a high susceptibility subtype manifested as bilateral rupture whose incidence and accelerated progression are strongly associated with gonad removal during the developmental period.

## Discussion

This report presents the first description of the gonadal correlates of estimated lifetime cruciate ligament survival in dogs. This lifetime study of the frequency and pattern of veterinarian-diagnosed CCL rupture in a breed at high risk for CCL rupture^8,18,26,27^ provides strong evidence that gonad retention during the first two years of life is associated with enhanced likelihood of cruciate ligament survival that persists over the next 11 years of life. The existence of a sensitive gonad exposure window that can jeopardize cruciate ligament survival is further supported by our observation that risk for CCL rupture was not influenced when gonad removal took place after the 24-month developmental period. The increased susceptibility conferred by early endocrine disruption was manifested as an increased likelihood of multiplicity, i.e., CCL rupture affecting both stifles. We found that risk of bilateral CCL rupture associated with gonad removal was partially mediated by early age at first rupture, linking early endocrine disruption with accelerated CCL failure. Moreover, risk reduction associated with the combination of short adult height and the avoidance of early gonad removal was additive. Importantly, the gonad-sensitive vulnerability to CCL rupture could not be explained by increased odds of becoming overweight or the presence of conformation defects or orthopedic conditions that affected the owner’s decision to select the timing of gonad removal. We contend that reinforcing gonad retention during the 24-month developmental period could lower the incidence of CCL rupture in this high-risk breed.

Though many disease outcomes are considered dichotomously (i.e., present or absent), an achievable goal of descriptive studies is to identify differences in phenotype, which can be readily recognized as clinical variants, that might enable surer footholds for exploring the genetic and non-genetic factors that confer disease susceptibility^28,29^. Analogous to the conceptualization of two distinct forms of diabetes mellitus in humans—type I and type II—our findings point to two distinct clinical CCL failure syndromes in Rottweilers. The first form is characterized by early, predominantly bilateral rupture that is sensitive to gonad removal during the developmental period. The second form consists of dogs with later rupture, typically unilateral, and insensitive to early gonad removal. We believe the first CCL failure syndrome represents the highest susceptibility based upon two separate measures of susceptibility: multiplicity (bilateral rupture) and early age at diagnosis. We suspect that the second form is to a larger extent associated with wear and tear, stochastic events, and other yet to be identified risk factors. A testable hypothesis—that these two patterns of CCL failure represent differential enrichment of CCL rupture risk alleles—could be explored. Defining the extent to which these two CCL failure syndromes differ with regard to joint chemistry and metabolism (e.g., inflammation)^3,30^ and radiographic indicators of conformation (e.g., tibial plateau angle, femoral anteversion angle)^31^ would be a logical extension of the current work that could catalyze additional, useful re-categorizations of canine cruciate ligament disease.

Previous reports on the association between early gonad removal and risk for developing CCL rupture have yielded inconsistent results^11,14,22,32,33^, with strongest associations reported in single-breed studies and studies where age at gonad removal (rather than gonad status at time of CCL rupture) was analyzed^15–17,20–22^. Seminal publications by Hart and colleagues showed an increased risk in many (but not all) breeds was associated with gonad removal during the first 12 months^15,17,20,21^. Our work here affirms their conclusion that less than 12 months of age is a CCL risk-associated gonad exposure window in Rottweilers^17^ and extends the sensitive gonad window to include the first 24 months of life.

Previous studies have documented earlier age at CCL rupture—a proxy for accelerated ligament failure—in dogs with bilateral rupture compared to dogs with unilateral rupture^33–36^. Muir et al.^35^ showed in Labrador Retrievers that later age at first rupture was associated with greater likelihood of contralateral cruciate ligament survival. The notion that large breed dogs, such as Rottweilers, may experience CCL rupture at an earlier age than smaller breeds has also been suggested by previous work^8,12,36^. However, none of the aforementioned studies examined whether earlier age at first rupture or risk for bilateral rupture was sensitive to gonad removal during the developmental period. Here, we documented a clear difference in average age at first rupture between Rottweilers with bilateral rupture (3.8 years) and unilateral rupture (9.9 years), reinforcing the differences reported in four multi-breed studies^33–36^. Importantly, our work extends the previous observations of earlier age at diagnosis among bilateral cases by showing that a particular phenotype—gonad removal during the developmental period—is associated with an apparent accelerated onset of first rupture. Avoidance of early endocrine disruption was associated with an estimated 5.5-year postponement of first CCL rupture in our study.

Our observation that age at first rupture appears to be a strong predictor of subsequent contralateral rupture may have important research implications for investigators seeking to establish breed-specific criteria for identifying individuals that are resistant to CCL rupture. In this cohort, all dogs in the bilateral rupture group had their index CCL rupture prior to 9 years of age; none of the 15 dogs with first rupture occurring after 9 years of age subsequently developed a second rupture during extended follow-up. Our work in Rottweilers suggests individuals that have not developed cruciate ligament rupture by 9 years exhibit a CCL resistant phenotype. This conclusion appears consistent with the approach used by investigators to assign non-cases (controls) in case-comparison studies of CCL rupture in Labrador Retrievers, in which 8-year old dogs with normal stifle examination and stifle radiographs free of degenerative changes bilaterally are categorized as non-cases^10^. Taken together, the findings from these two breeds support the notion that an age cut point could be practically developed as an effective way of excluding from research populations those dogs with lowest susceptibility to CCL rupture, thereby enhancing the likelihood of pinpointing genetic or non-genetic susceptibility factors.

Just how the sensitive window of gonad exposure might impact CCL rupture risk is the subject of increasing speculation. Removal of gonads may increase risk by delaying epiphyseal growth plate closure^37–40^. This delay in skeletal maturity might be associated with increased opportunity for errors in skeletal modeling that render the cranial cruciate ligament more susceptible to rupture. Two studies have demonstrated that the occurrence of increased tibial plateau angle (TPA), a phenotype that may be associated with increased risk for CCL rupture, is increased by gonad removal^41,42^. Although reported differences between TPA in cases and non-cases are inconsistent^32,34,35,43–47^, the finding that increased TPA may be a gonad-sensitive phenotype raises the possibility that other morphometric variables, such as intercondylar notch width^48^ or femoral anteversion angle^31^, may also be sensitive to early endocrine disruption.

Gonad removal during the developmental period may also exert deleterious effects on factors intrinsic to the cranial cruciate ligament and its articular environment. Independent of conformation, these intrinsic effects might undermine homeostasis, leading to accelerated failure. Collagen genes (e.g., *COL1A1*, *COL5A1*) expected to influence ligament structure and strength were among candidate genes associated with veterinarian-diagnosed CCL rupture in high-risk breeds^49^. Other genomic loci [c-type lectin receptors^10^, *SORCS2*^50^, *DOCK2*^45^] link CCL rupture risk with inflammatory/immune response, which is supported by clinical studies suggesting synovial inflammation precedes CCL rupture and may accelerate disease progression^3,5,35,51^. Estrogen has anti-inflammatory properties^52–54^ potentially rendering the stifle joint of gonad-deficient females a pro-inflammatory environment. Additional studies are needed to interrogate the complex influences of environmental factors, such as gonad removal, and genetic factors on key processes including collagen synthesis, fibril assembly and degradation that may influence disease onset and progression. Such integrative studies exploring CCL rupture susceptibility should attempt to reconcile why the apparent sensitivity to gonad removal is restricted to the developmental period. Search for a genetic basis for gonad sensitivity of CCL disease initiation and/or progression to CCL rupture, rather than the genetic basis for overall CCL rupture risk, could yield intriguing results. Risk alleles might increase CCL rupture susceptibility to early endocrine disruption in an exposure-trait specific manner, or have broader implications for health outcome than CCL rupture susceptibility, i.e. cancer susceptibility.

Dog fanciers have known for a long time that purebred dogs that undergo early gonad removal take on a different physical appearance—appearing taller, “stretched out”^37,55^. Rottweilers and other large and giant breeds achieve skeletal maturity during the second year of life^37,56,57^. Here, we documented that gonad removal during the 24-month developmental period was associated with increased likelihood of reaching tall height, though it is not clear whether these increments in additional height were directly attributable to early gonad removal. In humans, adult height is correlated with the risk of more than 30 diseases^58,59^. To our knowledge, the current study in dogs provides the first evidence that early gonad removal is associated with a degree of tallness that situates individuals at increased risk for an adverse adult health outcome, i.e., increased CCL rupture.

Across dog breeds, strong evidence supports larger body size as a significant risk factor for CCL rupture^8^. To our knowledge, however, the association between adult height and risk of CCL rupture in dogs has never been explored using individual-based data within the same breed. In our single-breed study, after accounting for sex-specific differences in height, avoidance of tall height was associated with a 70% reduction in CCL rupture. It is generally understood that height is strongly influenced by genetic factors^58–61^. *Ror2*, a gene expressed by growth plate chondrocytes and implicated in the patterning and growth of long bones^62^, has been linked to CCL rupture risk in Labrador Retrievers^45^. Taken together, our observed association between tall adult height and risk of CCL rupture may be gene-driven.

Yet a number of open questions accompany our findings on the relationship between adult height and risk of CCL rupture. It is unclear to what extent tall adult height directly contributes to an at-risk conformation or serves as a proxy for error-prone, maladaptive skeletal modeling taking place during a potentially prolonged time to reach skeletal maturity. In humans, longer time to reach adult height has been associated with riskier landing profiles during drop vertical jump test, which investigators postulated may lead to a higher susceptibility for anterior cruciate ligament injury^63^. Tall dogs that undergo gonad removal during the developmental period may very well represent the subset of Rottweilers that have the longest delay in reaching skeletal maturity, and this delay might affect their risk of CCL rupture. Indeed, in our study cohort, avoidance of the combination of tall adult height and early endocrine disruption was associated with a 93% risk reduction for any CCL rupture (Fig. 2). Based upon these findings, selective breeding to restrict adult height^38,60^ may offer a potential risk reduction strategy. The extent to which tall height in high-risk breeds is a phenotype that could be mitigated through non-genetic approaches (e.g., gonad retention, avoiding overnutrition) should also be explored.

In the Exceptional Aging in Rottweilers Study cohort, the strong gonad association with bilateral CCL rupture risk was independent of being overweight prior to orthopedic injury. These null results are consistent with the findings of Simpson and colleagues^16^, who estimated that only a small fraction (7%) of the gonadectomy influence on the incidence of non-traumatic orthopedic injury in Golden Retrievers was mediated by overweight/obesity. Our results are also congruent with four previous studies that showed no difference between bilateral and unilateral CCL rupture cases with respect to being overweight^32,34,35,64^. It should be noted, however, that our cohort may be poorly suited to investigate the relationship between being overweight and subsequent CCL rupture for at least two reasons. First, our findings rely upon owner-reported body condition rather than veterinarian-reported body condition score, which may misclassify or underestimate the number of dogs that were overweight or obese. Second, being overweight is not a phenotype that is highly prevalent among Rottweilers that reach exceptional longevity. Overall, only 12% of dogs in our cohort were overweight prior to CCL rupture, while 19% of dogs with lifelong resistance to CCL rupture were overweight. Perhaps, the low frequency of dogs that were overweight explains, in part, why we found no significant association between early endocrine disruption and being overweight, or between being overweight and CCL outcome. However, studying the gonad sensitivity of CCL outcome in a cohort such as this one may actually provide a potential advantage—the opportunity to map associations between gonad removal during the developmental period and CCL outcome in a population that is not confounded by a high prevalence of obesity. Indeed, we found that earlier age at first rupture—a surrogate for accelerated ligament failure—is a strong mediator of the effect of gonad removal on bilateral CCL rupture, without evidence that being overweight contributed to this outcome. Additional studies are clearly warranted to better define the extent to which body composition^65^ or obesity-associated inflammation contribute to inflammatory progression within the stifle joint, culminating in cruciate ligament failure^3,66,67^.

Our finding that the production of offspring may be associated with protection against subsequent development of an adverse health outcome mirrors results from women, in which a moderate level of parity is inversely associated with all-cause mortality^68^. The beneficial effect of parity on non-reproductive health outcomes may suggest the presence of other key variables that are differentially present in parous and non-parous females. We explored this possibility, but found no differences in adult height or body condition between parous and non-parous females in our cohort. It is plausible that biological mechanisms underlying the observed association may be related to epigenetic changes in gene expression. Parity has been associated with a deceleration of human aging^69^, and it has been proposed that chromatin modification (epigenetic memory) may enhance cardiovascular adaptation, resulting in improved reproductive outcomes after first pregnancy^70^. Future studies should attempt to replicate the association between the production of offspring and CCL rupture risk reduction.

There are limitations to our study. Some of the data were collected retrospectively, which may limit its accuracy and precision. Recall bias may limit the accuracy of information obtained during owner interviews and questionnaires. A key exposure variable, duration of gonad exposure, was not randomized among dogs in this cohort. Our study team collected from dog owners detailed information on lifetime gonad exposure. In a majority of cases, we maximized the accuracy of these data using the date of gonad removal surgery from medical records, and we excluded dogs from analysis if gonad status during the first 24 months of life could not be ascertained. Moreover, for each dog, we used telephone interview of owners to collect information on the specific reason for the decision to conduct gonadectomy. No previous study on canine CCL rupture has presented data on reason for gonadectomy. Including such information in our multivariate risk models enhances confidence that the observed associations between gonad removal and CCL outcome could not be attributed to conformation defects or orthopedic conditions (e.g., hip dysplasia, elbow dysplasia) that might precipitate early gonad removal, contribute to CCL rupture risk, thereby confounding interpretation. It should be noted that the elective decision to remove gonads in pet dogs is made based upon multifaceted considerations that extend beyond animal health, that may relate to the pet owner and the environment in which the dog lives. Additionally, the observational nature of our study provides information on associations, so our findings cannot affirm or refute causality.

Radiographs were not consistently available for review. The lack of radiographs as part of standardized assessment may be considered a study limitation, leading to underdetection and underdiagnosis of CCL rupture cases. Our research protocol did not include radiographic assessment because it was not practical to conduct a standardized radiographic assessment of dogs in this study population, i.e., elderly dogs living in 37 states and Canada. Instead, our in-home, near-end-of-life assessment of CCL status was limited to a standardized orthopedic evaluation of both stifles by a single experienced examiner. A standardized approach combining orthopedic examination with radiographic screening has been used to identify non-cases in genetic discovery studies^10^. It is possible that use of radiography in our study could have decreased the number of false negatives associated with our method of CCL outcome assignment. Use of radiography may have enabled the identification of unilateral CCL rupture in dogs that were categorized in our study as rupture resistant. Also, radiographs may have aided in identifying dogs with bilateral rupture incorrectly categorized in our study as unilateral rupture.

Because outcome misclassification is a persistent concern in clinical detection studies, there are several aspects of our investigative approach that were intended to promote accuracy of CCL outcome classification. First, all study subjects had a 13-year observation period corresponding to four years beyond breed-specific median lifespan. Because the study population was free of censoring for lost to follow up or death, false negatives that are inevitably associated with fragmented, incomplete follow-up were avoided. Second, examination of each dog was standardized (single experienced examiner) in non-acute stifles, in a non-stressful home environment, heightening the consistency of orthopedic examination results. Thirdly, end of life evaluation allows the natural history of degenerative CCL disease to play out—examiner’s role is to determine if any earlier-in-life prodromal inflammatory changes, early fiber disruption had progressed to CCL rupture-associated clinical lameness and associated physical findings not limited to cranial-caudal laxity, including periarticular changes consistent with chronic rupture. The limited healing capacity of the CCL may increase likelihood that partial tears initiated earlier in life progress to complete CCL rupture that would be more readily discernible from stifles without CCL rupture. Fourth, our late life examination revealed six dogs with occult CCL rupture in this cohort that would otherwise have been false negatives if our reporting was based solely on retrospective data collection from medical records, representing 10% of the total CCL rupture diagnoses in the cohort. It is perhaps notable that the relatively close concordance of our reported incidence rates of CCL rupture (148 cases per 10,000 dog-years at risk) with Swedish data on Rottweilers (127 cases per 10,000 dog-years at risk) (Table 1) is compatible with a moderate rate of underdiagnosis among dogs that we categorized as rupture resistant. Concordance between the relative proportion of bilateral versus unilateral cases in our cohort (43% bilateral) with published estimates in the literature^4,5^ (22–55% bilateral) may also suggest a moderate rate of underdiagnosis of contralateral rupture among dogs that we categorized as unilateral rupture.

Underdiagnosis leading to outcome misclassification may undermine confidence in reported associations. Because a certain degree of underdetection leading to misclassification is likely to have occurred using our study protocol, we evaluated the potential impact that underdiagnosis-driven CCL outcome misclassification might exert on the associations we reported in our primary analysis. We conducted random reassignment sensitivity analysis showing that even after reassignments of CCL outcome that mimic 20% false negative rate, 5% false positive rate up to 40% false negative rate, 5% false positive rate, our main findings remain robust: Gonad retention during the developmental period and short adult height are associated with sizeable risk reduction for bilateral CCL rupture (Supplementary Figure S2). This work on clinical correlates of lifetime cruciate ligament survival sets the stage for replication studies establishing lifetime CCL status using other methodologies, i.e., radiography, MRI, arthroscopy.

Finally, this cohort of North American dogs that reached exceptional longevity reflects a study population that differs from the general population of Rottweilers. As such, the uniqueness of this cohort indicates that the associations and effect sizes reported here may not be directly translatable to other populations. For example, further study is needed to determine if the sex-specific cut points of adult height used in our study translate into useful markers of CCL rupture risk differences in the general population of Rottweilers. In some analyses, small sample sizes limited multivariate modeling resulting in a potential lack of power to detect associations. However, our estimates of CCL rupture incidence^8^ (Table 1), the proportion of affected dogs with bilateral rupture^36^, and the highest sensitivity of CCL outcome to gonad removal during the first 6 months of life^17^ (Table 3) are highly comparable to previously reported findings in Rottweilers living in North America and Europe. Moreover, the work presented here may be considered a novel research approach to discover clues, to generate hypotheses regarding healthy lifespan by evaluating extrinsic factors, such as gonad removal, that may confer increased disease susceptibility in a long-lived population enriched for overall disease resistance^71^. With regard to opportunities for discovery, such populations may be ideally suited to detect the strength of signal that can emerge from comparing dogs that are clinically manifesting polar extremes in CCL rupture susceptibility—lifetime avoidance of veterinarian-diagnosed CCL rupture (*lowest susceptibility*) versus bilateral CCL rupture (*highest susceptibility*). Future studies capitalizing on this extreme comparison approach, rather than comparing dogs with any rupture to non-cases, may give rise to valuable new hypotheses.

### Conclusion

Our study provides the first description of clinical correlates of CCL rupture avoidance in dogs using an estimated lifetime cruciate ligament survival approach. Endocrine disruption during the 24-month developmental period is associated with adverse CCL outcomes, reflected by three measures of susceptibility—increased incidence rate, increased occurrence of bilateral rupture, and earlier age at first rupture. Based on these findings, together with the strong risk reduction associated with short adult height, we hypothesize that two risk reduction strategies—gonad retention and height restriction—initiated during the period of skeletal and neuromuscular development may be useful in supporting joint and ligament homeostasis, minimizing skeletal modeling errors and the advent of at-risk conformation changes, thereby enhancing cruciate ligament survival.

Moreover, we believe our demonstration of two distinct clinical syndromes of CCL failure within a single high-risk breed provides a germinative framework that can be used to establish a cumulative vulnerability index^72^ that could encompass polygenic risk score^73,74^ and radiographic conformation score^31,75^. Such a conceptual framework applied to other high-risk breeds may provide expanded and nuanced opportunities for better defining the contributing role of other factors such as obesity, inflammation, physical activity^76^, and dietary pattern in the pathogenesis of cruciate ligament failure.

## Methods

### Study population

The Exceptional Aging in Rottweilers Study (EARS) is an open registry, ambidirectional cohort study of client-owned Rottweiler dogs with exceptional longevity living in North America. Dogs enrolled into the study meet the following inclusion criteria: (1) validation of purebred status through American Kennel Club (AKC) registry database; (2) age of ≥ 13 years, which represents living more than 30% longer than the average lifespan of the breed^24,25^; and (3) owner willingness to provide information by questionnaire, medical records, and telephone interviews so that lifetime medical histories can be constructed. In addition to this retrospective collection of information, there is a prospective phase of data acquisition, which includes collection and biobanking of clinical samples and DNA, construction of frailty scores through sequential telephone interviews, and collection of tissue samples at necropsy. Since 2003, more than 400 Rottweilers with exceptional longevity have been enrolled. Eligible for the current analysis are the 127 dogs with more intensive data collection including a standardized orthopedic examination performed by a single examiner during a four-hour home visit, allowing for a standardized ascertainment of estimated lifetime cruciate ligament survival. Supplemental Table S11 shows that the 127 eligible dogs did not differ from the other 298 dogs enrolled in EARS that did not receive in-home examination in terms of geographic residence, female : male ratio, duration of gonad exposure, prevalence of overweight body condition, or cause of death. Four of the 127 eligible dogs are excluded from the current report; in two dogs, an accurate assessment of CCL status could not be determined at the time of examination (previous fracture of tibial tuberosity, soft tissue sarcoma of stifle region). In two other dogs, information on age at gonad removal was insufficient to conduct gonad exposure window analyses. Thus, the current cohort analysis is limited to 123 dogs that were deceased at the time of this analysis.

Enrollment in EARS is owner-driven, initiated by owner contact with research staff members regarding a dog suspected of being study-eligible. Outreach to veterinarians, dog owners, and dog breeders was used to enhance study visibility and heighten awareness of the need for longevity research in pet dogs through speaking engagements at national veterinary conferences and local and national breed club meetings. A one-page study brochure was introduced at these events and made available at the study website. Enrollment of dogs living in North America was not limited to a particular geographic proximity to investigators or to urban-suburban-rural setting. The ability to enroll unregistered dogs was achieved through AKC registry confirmation of purebred status of both dam and sire or purebred status of littermates. There was no monetary incentive for study participation; no provisions were made for participants to receive free or discounted treatment services.

The 123 dogs of this report represent the subset of dogs in EARS that had in-home examinations. Dogs in the EARS study are not enrolled based upon whether their medical history indicates that they have been affected by any medical condition, such as cruciate ligament rupture. Selection for in-home visit was not based upon investigators’ prior knowledge of exposure variables—height, gonad exposure, body condition, parity—that were evaluated in the current study or prior knowledge of orthopedic abnormalities. Instead, data collection protocol during the in-home visit was designed to collect a wide variety of measurements and observations related to a broad range of clinical phenotypes (e.g., hearing loss, appetite, neurologic function) to construct a rich snapshot of Rottweilers reaching exceptional longevity. Study eligibility criteria required the examiner to have sufficient familiarity with medical histories to aid the examiner in the detailed and complete collection of information from each dog. At the time of home visit data collection, the investigative team held no formal hypothesis related to pathogenetic factors and canine CCL rupture. Pet owners at the time of questionnaire completion and in-home examination were similarly unaware of any subsequent CCL rupture-related inquiry because the stated goal of the research was to promote a scientific understanding of longevity determinants, without targeting any particular disease condition.

### Ascertainment of cranial cruciate ligament (CCL) status and estimation of lifetime CCL survival

Lifetime medical histories were combined with results of a standardized near-end-of-life orthopedic assessment to quantify the number of veterinarian-diagnosed CCL ruptures in this exceptionally long-lived cohort of Rottweilers, thereby providing an estimate of lifetime CCL survival in a high-risk breed. The proportion of dogs with estimated lifetime CCL survival was calculated as:

$$\begin{gathered} \hfill \\ \frac{{\left[ {\text{Total Dogs in Cohort}} \right]{\text{ minus }}\left[ {{\text{Number of Dogs with Veterinarian}} - {\text{diagnosed CCL Rupture}}} \right]}}{{\left[ {\text{Total Dogs in Cohort}} \right]}} \hfill \\ \end{gathered}$$

After study enrollment, each dog underwent standardized assessment of both stifle joints by a single veterinary specialist (Diplomate ACVS, 1990) possessing more than 20 years of experience in the clinical diagnosis and management of canine CCL rupture. The standardized near-end-of-life exam was used to provide internal consistency in assigning lifetime CCL status, to increase confidence in confirming veterinarian-diagnosed CCL rupture among dogs with non-surgically treated stifles, and to identify dogs with previously undiagnosed CCL rupture. All stifle examinations were performed in the dog’s home, under quiet conditions, with the dog in lateral recumbency on a comfortable floor surface. Because none of the dogs were suffering from an acute injury to the stifle, dogs did not display the guarding of stifle movement and reticence to examination that can challenge reliable assessment of drawer sign and other physical aspects in non-sedated, acutely injured patients.

There were four diagnostic paths by which dogs in the cohort were assigned as having veterinarian-diagnosed CCL rupture (Supplementary Figure S1). In 24 dogs in this cohort, diagnosis of 38 CCL ruptures was made at the time of open stifle surgery. Path of diagnosis in the 22 non-surgical cases of CCL rupture in this cohort reflected one of 3 criterial methods: (1) Eight dogs had a clinical history of acute stifle lameness/pain associated with diagnosis of suspected CCL rupture, with excess cranial-caudal laxity confirmed at the time of standardized near-end-of-life orthopedic examination; (2) Eight dogs had a history of stifle lameness associated with suspected CCL rupture diagnosis, but without dramatic increase in laxity but substantial periarticular fibrosis, crepitus consistent with chronic CCL rupture identified at the time of the near-end-of-life orthopedic examination; (3) Six dogs had no clinical history of an event suggestive of CCL rupture reported by owner or veterinarian (so-called occult), with assignment to a CCL rupture group based upon standardized near-end-of-life orthopedic exam revealing excessive cranial-caudal laxity and/or extensive periarticular fibrosis.

Supplementary Figure S1 depicts the assignment path and outcome disposition of each dog in the cohort. Notably, 81 of 123 dogs in the cohort had no medical history of stifle injury or stifle surgery, and on examination of both stifles showed no pain on joint flexion or extension, no joint effusion or thickening of periarticular tissues, and negative cranial-caudal instability during cranial drawer and tibial thrust tests. These 81 dogs provided a lifelong CCL rupture-resistant group that could be compared with the other 42 dogs in this cohort that had veterinarian-diagnosed CCL rupture. Eighteen dogs were assigned to the bilateral rupture group. Twenty-four dogs were assigned to the unilateral rupture group; nine dogs with periarticular fibrosis-based unilateral CCL rupture diagnosis did not differ from the 15 dogs that had either open surgery or laxity-based diagnosis of unilateral CCL rupture on the basis of sex, adult height, gonad exposure, age at rupture, or overweight body condition (Supplementary Table S12). Thus, in our primary data analyses, three categories of CCL outcome were used: RESISTANT (n = 81 dogs); UNILATERAL RUPTURE (n = 24 dogs) and BILATERAL RUPTURE (n = 18 dogs).

Age at cruciate ligament rupture was recorded using information from questionnaire and medical records. For dogs that underwent surgical repair of CCL rupture, age at rupture was recorded as the age at surgery, i.e., age at which the diagnosis was visually confirmed. This approach was considered reasonable because the interval from onset of clinical lameness to surgery was typically brief (median interval = 2 weeks, range 2 days–40 weeks; only two dogs had interval greater than 8 weeks; data not available for seven dogs). For dogs that did not undergo surgical repair, age at rupture was considered as the age at visit to a veterinary clinic with stifle lameness attributable to CCL injury. Age at rupture was not available for six occult CCL ruptures detected at the standardized near-end-of-life examination that were previously unnoticed by owners or veterinarians; four ruptures occurred in dogs in the unilateral group and two ruptures in dogs in the bilateral group representing a CCL rupture in the second/contralateral stifle.

### Exposure variables

#### Gonad exposure

Duration of lifetime gonad exposure (years) was measured by calculating age at gonad removal. In most instances, age at gonad removal was obtained by comparing date of birth and date of gonad removal surgery found in medical records. When medical record validation of gonad removal date was not available, the date of gonad removal provided in the owner questionnaire was used. If date was recorded in the questionnaire as month/year, the 15th day of the month was used to estimate age at gonad removal.

#### Reason for gonad removal

A standardized telephone interview was used to collect information to categorize the reason for gonad removal from owners of the 109 dogs that underwent gonad removal: (1) no breeding or no further breeding; (2) treatment or prevention of reproductive or other medical problems (e.g., prostatic disease, mammary gland neoplasia, behavioral issues); (3) substandard conformation (e.g., dental malocclusion, hair color, hip or elbow dysplasia); and (4) treatment of pyometra in females. Fourteen males, no females remained intact throughout their lifetime. No dogs underwent gonad removal for the treatment of CCL rupture. This interview enabled investigators to identify any dogs whose reason for gonad removal was a conformational defect or orthopedic condition (e.g., hip dysplasia, elbow dysplasia) that may have potentially influenced CCL rupture risk, thereby affecting the reported relationship between age at gonad removal and CCL outcome.

#### Adult height

Height was measured at the top of the shoulder (withers) during the standardized in-home examination. Duplicate measurements were made by a single examiner using a retractable, non-stretchable measuring tape. Each dog was measured in a normal standing position. If measurements made in duplicate lacked consistency, a third measurement was made using the same technique and the median value recorded as adult height. Median age of dogs in this cohort at the time of height measurement was 13.5 years. A previous study in dogs showed no aging-related “shrinkage” (as reported in elderly humans) in height measured at the shoulder at 3–7.9 years, 8–12.9 years, and 13 + years^56^.

#### Body condition

Owner-reported body condition of each dog was collected retrospectively by questionnaire. Owners selected one of four possible body conditions [underweight; ideal; overweight; markedly overweight (obese)] for three different periods during the life course [(pre-adult (6–9 months of age); adult, (4–6 years of age); and older adult (more than 7 years)]. Body condition responses were temporally matched with age at CCL rupture so that dogs could be dichotomized into: (1) overweight prior to CCL rupture (i.e., precedent overweight); or (2) not overweight prior to CCL rupture. Nine dogs in this cohort scored as underweight were grouped together with dogs with reported ideal body condition to create the “not overweight” category. Body weight was not consistently available and therefore not used in any analysis.

#### Parity

Detailed information on lifetime reproductive history for each female was obtained through questionnaire and telephone interview with owners. Date at whelping, number of litters and live puppies were obtained. For this analysis, parity was categorized as: (1) parous (producing offspring); or (2) non-parous (no offspring) based upon these data.

### Statistical analysis

Data analyses were performed using STATA Version 17 (Stata Corp., College Station, TX, USA). Statistical significance was defined as *p* < 0.05 and all tests were two sided. Descriptive characteristics for groups were expressed as medians (IQR, range) or proportions (%) and compared using Chi-square and t tests. Descriptive data were analyzed for male–female differences (Supplementary Table S1). Cumulative frequency distribution function was used to visually compare dogs in the unilateral and bilateral rupture groups with respect to age at gonad removal, adult height, and age at first rupture (Fig. 4). For the calculation of incidence rates, dog-years at risk (DYAR) were based on the 13-year study observation period and the time until first rupture. To directly compare the estimates of incidence rate in this cohort with the only published estimates of the incidence rate of CCL rupture in dogs, we expressed incidence rates per 10,000 DYAR before and after adjustment for early gonad removal and age restrictions in enrollment observed within the Swedish insured dog population^8^ (Table 1). Calculation of incidence rates excluding males and females with gonad removal during the developmental period enabled an estimate of a female : male risk ratio free of the confounding effect of gonad removal.

To determine the association between estimated lifetime cruciate ligament survival and exposure variables, Kaplan–Meier survival analysis was used to compare subgroups stratified by: sex (male, female), adult height (short, tall), gonad exposure (brief exposure, longer exposure), body condition (precedent overweight, not overweight) (Fig. 1). Differences were tested for significance using log-rank test. For these analyses, lifetime CCL survival was operationalized as survival of both cranial cruciate ligaments at 13 years, because each dog lived to 13 years of age. In survival analyses, missing values for four dogs with unknown age at first CCL rupture were imputed using nearest neighbor imputation^77^ informed by the observed pattern between duration of gonad exposure and age at first rupture. One dog that experienced CCL rupture after 13 years of age was coded in this analysis as free of CCL rupture at 13.0 years. Because differences in both adult height and gonad exposure had significant associations with estimated lifetime cruciate ligament survival, a four-category variable method was used to evaluate for additive effects (Fig. 2).

To estimate the risk of CCL rupture associated with each variable, Cox proportional hazards model was used to generate hazard ratios. The proportional hazards assumption was not violated in any of the Cox models. Selection of risk variables in final models was guided by likelihood ratio test for fitness and by practical consideration of necessary variables. No censoring prior to end of the study observation period occurred since no dogs were lost to follow-up or died between 0 and 13.0 years of age. Distribution of continuous variables in the data set (e.g., adult height, gonad exposure) were evaluated using scatterplots and did not yield any outliers or influential points. For each exposure variable, risks were estimated for three possible CCL rupture outcomes: (1) any rupture; (2) unilateral rupture; and (3) bilateral rupture. Cox proportional hazard models for any CCL rupture and for unilateral CCL rupture were used to model time to first rupture. Cox proportional hazard models for bilateral CCL rupture modeled time to second rupture after nearest neighbor imputation of two missing values for age at second rupture (Table 4). Non-adjusted and adjusted (sex, age at CCL status ascertainment, reason for gonad removal) hazard ratios (HR) and 95% confidence intervals (95% CI) were reported. In these analyses, reference groups (HR = 1.0) were assigned so that hazard ratios of the other exposure groups reflected an estimate of risk reduction.

Adult height was tested in the Cox regression model as a continuous variable and also dichotomized to express tallness. Because height is a sexually dimorphic phenotype, male and female dogs were assigned to “short” versus “tall” categories using sex-specific cut points (61.0 cm for females, 64.8 cm for males) based upon breed-specific standard values^78^ and the distribution of adult height within the males and females of this study cohort (Supplementary Figure S4 and Supplementary Table S4). To examine the association between age at gonad removal and adult height, dogs were segregated into four gonad exposure groups (≤ 6 months, 6.01–12 months, 12.01–24 months, > 24 months), then median differences between measured height and sex-specific breed standard adult height were compared using non-parametric independent samples median test (Supplementary Table S5).

Cox regression model was used to estimate risk of CCL rupture associated with being overweight prior to CCL rupture. None of the dogs with bilateral rupture had precedent overweight. However, five dogs with unilateral rupture were overweight based upon owner reporting. Because an owner could only report a single body condition score per life course period, and a dog’s body condition could change during the life course period in which CCL rupture diagnosis occurred, data were handled to maximize the number of dogs coded as precedent overweight, thereby maximizing likelihood of detecting an association between precedent overweight and CCL rupture.

Gonad exposure was tested as a risk variable in the Cox regression model using three different cut points: (1) ≤ 6 months versus > 6 months; (2) ≤ 12 months versus > 12 months; and (3) ≤ 24 months versus > 24 months. In each instance, the group with shorter gonad exposure was selected as the reference group so hazard ratios would estimate CCL rupture risk reduction associated with gonad retention. Risk associated with age at gonad removal were adjusted for reason for gonad removal expressed as a binary variable (conformational/orthopedic defect versus all other reasons for gonad removal). For each of these three gonad exposure categories, postponement of first CCL rupture was calculated by subtracting median age at first rupture in the group that underwent early gonad removal from median age at first rupture in dogs that avoided gonad removal (Supplementary Table S8). To analyze the relationship between gonad removal and CCL outcome in more detail, a gonad exposure window analysis was performed (Table 3). Dogs were stratified into exclusive groups reflecting three different time windows of gonad removal during the developmental period: (1) ≤ 6 months (n = 11 dogs); (2) 6.01–12 months (n = 10 dogs); and (3) 12.01–24 months (n = 12 dogs). Dogs that retained their gonads for more than 24 months served as the reference group in this comparison.

To identify factors that were significantly associated with age at first CCL rupture (early versus late based upon median cut point), we used logistic regression in the 38 dogs affected with unilateral or bilateral cruciate ligament rupture (Supplementary Table S7). Next, in these 38 dogs, we determined if later age at first rupture was associated with significant risk reduction for bilateral rupture, using logistic regression that included gonad exposure in the model (Supplementary Table S9). Among the subset of dogs with bilateral CCL rupture, we tested whether earlier age at first rupture was associated with shorter interval to second (contralateral) rupture using Spearman rank test (Supplementary Figure S3). In 34 dogs with CCL rupture that had risk exposure dyads of EARLY GONAD REMOVAL—EARLY RUPTURE (high risk phenotype) or GONAD RETENTION > 24 MONTHS—LATE RUPTURE (lower risk phenotype), the percent concordance between the high risk phenotype and bilateral CCL rupture outcome and percent concordance between the lower risk phenotype with unilateral CCL rupture outcome was combined to calculate the overall predictive accuracy of these two risk dyads for distinguishing between dogs with bilateral versus unilateral rupture.

Because the strong association between gonad removal and CCL rupture suggested that cruciate ligament disease may be sensitive to alterations in sex hormones, we investigated whether the production of offspring (parity) was associated with CCL rupture risk in the subgroup of tall females that retained their ovaries and reached reproductive maturity. The testing of parity in the Cox proportional hazards model of CCL rupture (Supplementary Table S10) was limited to 26 females that reached reproductive maturity (ovary exposure more than 24 months) and were tall, because of the infrequent occurrence of CCL rupture (only 4%) in short reproductively mature females.

Mediation analysis using generalized structural equation modeling^79^ was used to estimate the extent to which variables (height, age at first rupture, overweight) might mediate the impact of gonad removal during the developmental period on the risk of bilateral CCL rupture. Direct, indirect, and total effects of gonad removal on bilateral CCL rupture were estimated. Mediation was assumed to have occurred when the indirect effect was statistically significantly different from zero. Following the suggestion of MacKinnon, Warsi, and Dwyer^80^, we calculated the effect size for mediation by computing the proportion of total effect mediated, the ratio between indirect effect and total effect (Fig. 3).

Finally, to assess the potential impact that CCL rupture underdetection might exert on the associations found in our primary analysis, sensitivity analysis^81^ was performed. The association between CCL rupture and two potential risk reduction phenotypes—short adult height, avoidance of gonad removal during the developmental period—was re-analyzed after random reassignment of CCL outcome (Resistant, Unilateral Rupture, Bilateral Rupture) to mimic plausible levels of underdetection and misclassification in the cohort. Using a publicly available random number generator (RANDOM.org), random, category-specific outcome reassignment of 23 dogs was used to generate a CCL outcome distribution that simulated a 20% false negative rate, 5% false positive rate in our original data set. Reassignment of CCL outcome was as follows: Sixteen dogs were moved (“movers”) from Resistant to Unilateral Rupture group and 5 dogs were moved from Unilateral to Bilateral group to mimic 20% false negative rate; one dog was moved from Unilateral to Resistant group and one dog was moved from Bilateral to Unilateral group to mimic 5% false positive rate (Model 2, Supplementary Figure S2, panel A). In this sensitivity analysis, there was no reassignment of risk variables. Logistic regression was used to determine pre- and post-reassignment odds ratio (OR) and 95% CI so that changes in effect size and statistical significance of selected risk phenotype—CCL outcome associations could be estimated. Risk was estimated using logistic regression, rather than Cox proportional hazards modeling, because it obviated the need for imputing missing values for age at first rupture for Resistant movers. Separate analyses were conducted for the outcomes of Bilateral Rupture and for Unilateral Rupture before and after random CCL outcome reassignment (Supplementary Figure S2, panel B). To assist in interpretation of results, the proportion of dogs harboring high-risk phenotypes (tall height, EED_24months_) was depicted across the resultant models of outcome misclassification (Supplementary Figure S2, panel C). The proportion of dogs harboring high-risk phenotypes was also depicted for movers and for dogs in the original CCL outcome categories (Supplementary Figure S2, panel D). Models were also created to mimic more severe outcome misclassification of up to 40% false negative rate, 5% false positive rate (Models 3 and 4, Supplementary Figure S2).

### Ethics statement

This study was reviewed and approved by the Animal Care and Use Committee of Purdue University (protocol #95-026) and Gerald P. Murphy Cancer Foundation (protocol #15-001). All procedures were conducted in accordance with relevant guidelines and regulations. Written informed consent was obtained from owners prior to the collection of biological samples from study animals.

### Supplementary Information

Supplementary Information.

## Data Availability

The data generated or analyzed during this study are included in this published article and its Supplementary Information files.

## Abbreviations

CCL: Cranial cruciate ligament
EARS: Exceptional Aging in Rottweilers Study
EED_24m_: Early endocrine disruption (EED) caused by gonad removal during the developmental period, i.e., the first 24 months_(24__ m__)_ of life
