# Supplementary material for: Correlates of estimated lifetime cruciate ligament survival inform potential rupture risk reduction strategies: findings from the Exceptional Aging in Rottweilers Study
Source: Sci Rep. 2023 Aug 25;13:13920. doi: 10.1038/s41598-023-39288-y (PMC10457323; doi:10.1038/s41598-023-39288-y)
Supplement: Supplementary file 1 — Supplementary Information. [file 41598_2023_39288_MOESM1_ESM.docx]

Supplementary Material

Correlates of Estimated Lifetime Cruciate Ligament Survival
Inform Potential Rupture Risk Reduction Strategies:
Findings from the Exceptional Aging in Rottweilers Study

David J. Waters, Rong Fu, Andres E. Carrillo, Emily C. Chiang, Aimee H. Maras, Seema S. Kengeri & Cheri L. Suckow

| **Variable** | **Females (n=76)** | **Males (n=47)** | **p-value**† |
| --- | --- | --- | --- |
| **Lifetime Cranial Cruciate Ligament (CCL) Status** |  |  | 0.32 |
| Resistant, n (%) | 48 (63.2) | 33 (70.2) |  |
| Unilateral rupture, n (%) | 14 (18.4) | 10 (21.3) |  |
| Bilateral rupture, n (%) | 14 (18.4) | 4 (8.5) |  |
| **Age at First Rupture,** median (IQR), in years†† | 5.4 (3.3, 9.4) | 9.9 (5.4, 11.2) | 0.02 |
| **Age at Gonad Removal,** median (IQR), in years | 4.4 (1.1, 6.8) | 6.8 (2.5, 13.5) | 0.05 |
| **Adult Height,** median (IQR), in centimeters | 61.0 (58.4, 61.0) | 63.5 (63.5, 64.8) | < 0.001 |
| **Body Condition** |  |  |  |
| Precedent Overweight, n (%) | 14 (18.4) | 6 (12.8) | 0.41 |
| **Age at CCL Status Ascertainment,** median (IQR), in years | 13.5 (13.3, 13.9) | 13.5 (13.2, 13.8) | 0.92 |

**Supplementary Table S1.** Summary of characteristics of 123 dogs in the Exceptional Aging in Rottweilers (EARS) cohort: Comparison of females vs. males. †p-values based upon chi-square test statistic for categorical variables, t test for continuous variables. ††Age at first rupture is reported for 28 females and 14 males with CCL rupture. CCL = cranial cruciate ligament; IQR = interquartile range.

| **Variable** | **Resistant**  **(n=81 dogs)** | **Unilateral rupture**  **(n=24 dogs)** | **Bilateral rupture**  **(n=18 dogs)** |
| --- | --- | --- | --- |
| **Age at First CCL Rupture,** in years† | --- | 10.0 (6.8, 11.3) | 3.8 (2.5, 5.2) |
| **Sex (female),** n (%) | 48 (59.3) | 14 (58.3) | 14 (77.8) |
| **Age at Gonad Removal,** in years† | 6.1 (2.8, 9.0) | 4.5 (2.2, 7.7) | 0.5 (0.5, 1.5) |
| **Reason for Gonad Removal,** n (%) |  |  |  |
| Conformation defects/Orthopedic conditions | 6 (8.7) | 0 (0.0) | 1 (5.9) |
| Other reasons | 63 (91.3) | 23 (100.0) | 16 (94.1) |
| **Number of Dogs with Intact Gonads Throughout Lifetime (females, males)** | 0, 12 | 0, 1 | 0, 1 |
| **Reproductive Status in 53 Females with >24 months Gonad Exposure,** n (%) |  |  |  |
| Parous | 19 (45.2) | 1 (11.1) | 1 (50.0) |
| Non-parous | 23 (54.8) | 8 (88.9) | 1 (50.0) |
| **Adult Height,** in centimeters† |  |  |  |
| Females | 59.7 (58.4, 61.0) | 61.0 (61.0, 62.2) | 61.0 (60.6, 64.8) |
| Males | 63.5 (62.2, 64.8) | 64.8 (63.5, 66.4) | 66.0 (61.9, 68.3) |
| **Body Condition** |  |  |  |
| Precedent Overweight, n (%) | 15 (18.5) | 5 (20.8) | 0 (0.0) |
| **Age at CCL Status Ascertainment,** in years† | 13.5 (13.3, 13.8) | 13.5 (13.2, 13.8) | 13.3 (13.2, 13.9) |

**Supplementary Table S2.** Characteristics of 123 dogs in the Exceptional Aging in Rottweilers Study cohort stratified by three categories of cranial cruciate ligament (CCL) outcome: Resistant; Unilateral Rupture; Bilateral Rupture. CCL = cranial cruciate ligament.  †Values for continuous variables are expressed as median (interquartile range).

| **Gonad Exposure** | **n** | **Hazard Ratio** | **95% CI** | **p-value** |
| --- | --- | --- | --- | --- |
| **Intact Gonads at 24 months (n=90)** | | | | |
| Intact >48 months | 70 | 1.0 (ref) |  |  |
| Gonad removal at 24.01 – 48 months | 20 | 0.78 | 0.26-2.32 | 0.65 |
| **Intact Gonads at 48 months (n=70)** | | | | |
| Intact >72 months | 51 | 1.0 (ref) |  |  |
| Gonad removal at 48.01 – 72 months | 19 | 2.19 | 0.83-5.75 | 0.11 |
| **Intact Gonads at 72 months (n=51)** | | | | |
| Intact >96 months | 30 | 1.0 (ref) |  |  |
| Gonad removal at 72.01 – 96 months | 21 | 1.00 | 0.28-3.55 | 0.99 |

**Supplementary Table S3.** Gonad removal versus gonad retention after 24 months of age is not associated with risk of cranial cruciate ligament (CCL) rupture. Unadjusted hazard ratio (HR) and 95% confidence interval (95% CI) generated using Cox proportional hazard model. Analysis considers dogs that had intact gonads at three different ages (24 months, 48 months, 72 months). Hazard ratio (HR) reflects the risk of any CCL rupture in dogs that experienced gonad removal during the next 24-month interval compared to dogs retaining gonads during that interval. n = number of dogs. ref = reference group.

| **Height Category** | **Males & Females (n=123)** | | | **Females (n=76)** | | | **Males (n=47)** | | |
| --- | --- | --- | --- | --- | --- | --- | --- | --- | --- |
|  | n | HR (95% CI) | p-value | n | HR (95% CI) | p-value | n | HR (95% CI) | p-value |
| **Dichotomous** | | | | | | | | | |
| Tall | 61 | 1.0 (ref) |  | 41 | 1.0 (ref) |  | 20 | 1.0 (ref) |  |
| Short | 62 | 0.23 (0.11-0.47) | <0.001 | 35 | 0.19 (0.07-0.50) | 0.001 | 27 | 0.33 (0.11-0.98) | 0.05 |
| **Tertiles** | | | | | | | | | |
| Tall | 37 | 1.0 (ref) |  | 17 | 1.0 (ref) |  | 20 | 1.0 (ref) |  |
| Middle | 51 | 0.60 (0.32-1.15) | 0.12 | 34 | 0.60 (0.26-1.34) | 0.21 | 17 | 0.42 (0.13-1.35) | 0.15 |
| Short | 35 | 0.21 (0.08-0.57) | 0.002 | 25 | 0.19 (0.06-0.61) | 0.005 | 10 | 0.18 (0.02-1.40) | 0.10 |

**Supplementary Table S4.** Association between adult height and risk of any cranial cruciate ligament (CCL) rupture. Unadjusted hazard ratio (HR) and 95% confidence interval (95% CI) generated using Cox proportional hazards model. Dichotomous model uses sex-specific cut points for short versus tall (TALL females >61.0 cm; TALL males >64.8 cm). Female tertiles: Tall: >61.0 cm | Middle: 59.7 – 61.0 cm | Short: <58.4 cm; Male tertiles: Tall: >64.8 cm | Middle: 63.5 – 64.1 cm | Short: <62.2 cm. n = number of dogs; ref = reference group.

|  | **Age at Gonad Removal (Duration of Gonad Exposure)** | | | |
| --- | --- | --- | --- | --- |
|  | **≤ 6 months (n=11)** | **6.01 – 12 months (n=10)** | **12.01 – 24 months (n=12)** | **˃ 24 months (n=90)** |
| Median Height (cm) | 64.8 | 61.0 | 62.2 | 61.0 |
| Median ∆ from sex-specific standard (cm) | 2.5 | 0.0* | 0.0* | -0.6* |

**Supplementary Table S5.** Association between age at gonad removal and adult height in the study cohort (n = 123). A nonparametric independent samples median test was conducted to determine if there was a difference between four gonad exposure groups (≤ 6 months; 6.01-12 months; 12.01-24 months; and ˃ 24 months) in the median difference (delta) between measured height and published sex-specific height standard (males: 64.8 cm; females: 59.7 cm). Pairwise comparisons of differences from sex-specific standard adult height revealed that the dogs in the group with ≤ 6 months gonad exposure were significantly taller than the 6.01-12 months gonad exposure group (p = .022), taller than the 12.01-24 months gonad exposure group (p = .002), and taller than the ˃ 24 months gonad exposure group (p = .032). Reported significance values (p-values) were adjusted by the Bonferroni correction for multiple tests. *indicates significant difference compared to ≤ 6 months gonad exposure group (p < .05). delta (∆) = difference between measured height and sex-specific standard adult height.

|  | **Gonad Exposure** | **Hazard Ratio** | **95% CI** | **p-value** |
| --- | --- | --- | --- | --- |
|  |  | | | |
|  | **> 24 months**  **(n=72)** | 1.0 (ref) | – | – |
|  |  | | | |
| **Windows During Developmental Period** | **12.01 – 24 months**  **(n=7)** | 7.13 | 1.19-42.70 | 0.03 |
|  |  | 6.71* | 1.11-40.63 | 0.04 |
|  |  | | | |
|  | **6.01 – 12 months**  **(n=9)** | 12.71 | 2.82-57.24 | 0.001 |
|  |  | 10.50* | 2.30-48.00 | 0.002 |
|  |  | | | |
|  | **≤ 6 months**  **(n=11)** | 36.33 | 9.61-137.31 | <0.001 |
|  |  | 24.80* | 6.30-97.73 | <0.001 |

**Supplementary Table S6.** Association between age at gonad removal and risk for bilateral cranial cruciate ligament (CCL) rupture: An analysis of three distinct windows of gonad exposure (< 6 months, 6.01-12 months, 12.01-24 months) during the developmental period. For each developmental window, hazard ratio (HR) and 95% confidence interval (95% CI) for bilateral CCL rupture were generated using Cox proportional hazards modeling using dogs with > 24 months gonad exposure as reference (ref) group (HR = 1.0). Note wide confidence intervals as consequence of small subgroup comparisons. *Represents the adjusted hazard ratio (HR) including sex and adult height in multivariate analysis.

| **Variable** | **Unadjusted OR (95% CI)** | **p-value** | **Adjusted OR**  **(95% CI)** | **p-value** |
| --- | --- | --- | --- | --- |
| **Sex** | | | | |
| Male | 1.0 (ref) |  |  |  |
| Female | 2.73 (0.65-11.40) | 0.17 | 2.20 (0.35-13.73) | 0.40 |
| **Gonad Exposure** | | | | |
| >24 months | 1.0 (ref) |  |  |  |
| <24 months | 14.93 (3.01-74.07) | 0.001 | 13.99 (2.67-73.30) | 0.002 |
| **Adult Height** | | | | |
| Short | 1.0 (ref) |  |  |  |
| Tall | 3.11 (0.66-14.60) | 0.15 | 2.63 (0.37-18.81) | 0.34 |

**Supplementary Table S7.** Factors associated with early age at first cranial cruciate ligament (CCL) rupture. Dogs with CCL rupture were divided into early (<6 years) versus late (>6 years) age at first rupture based upon the median age at first rupture (n = 38 dogs). Unadjusted and adjusted odd ratios (OR) and 95% confidence interval (95% CI) were generated using logistic regression. Gonad exposure categories: >24 months = avoidance of gonad removal during the first 24 months of life; <24 months = early endocrine disruption. Tall height = females >61.0 cm, males >64.8 cm. ref = reference group.

| **Variable** | **Gonad Removal <6 months of age (EED_6months_)** | **Avoidance of Gonad Removal during first 6 months of life** | **Gonad Removal <12 months of age (EED_12months_)** | **Avoidance of Gonad Removal during first 12 months of life** | **Gonad Removal <24 months of age (EED_24months_)** | **Avoidance of Gonad Removal during first 24 months of life** |
| --- | --- | --- | --- | --- | --- | --- |
| Number of dogs | 9 | 29 | 14 | 24 | 21 | 17 |
| Age at First Rupture in years, median (IQR) | 3.2  (2.4, 4.2) | 8.7  (4.7, 10.3) | 3.9  (2.5, 4.8) | 9.6  (5.8, 11.1) | 4.3  (2.9, 6.5) | 9.7  (6.4, 11.7) |
| Postponement,  in years | 5.5 | | 5.7 | | 5.4 | |

**Supplementary Table S8.** Avoidance of early endocrine disruption (EED) is associated with postponement of first cranial cruciate ligament (CCL) rupture (n = 38 dogs). Median age at first rupture is compared between dogs with EED versus longer duration of gonad exposure using three different cut points of gonad exposure: ≤6 months vs. >6 months; ≤12 months vs. >12 months; ≤24 months vs. >24 months. For each gonad exposure cut point, postponement is calculated by subtracting median age at first rupture in EED group from median age at first rupture in avoidance of gonad removal group. Postponement of CCL rupture associated with avoidance of gonad removal is consistent across each of the three gonad exposure cut points during the 24-month developmental period. EED_24monts_ = early endocrine disruption (EED) caused by gonad removal during the developmental period, i.e., the first 24 months_(24months)_ of life. IQR = interquartile range.

| **Variable** | **Unadjusted OR (95% CI)** | **p-value** | **Adjusted OR**  **(95% CI)** | **p-value** |
| --- | --- | --- | --- | --- |
| **Gonad Exposure** | | | | |
| <24 months | 1.0 (ref) |  |  |  |
| >24 months | 0.09 (0.02-0.41) | 0.002 | 0.14 (0.02-0.96) | 0.04 |
| **Age at First Rupture** | | | | |
| <4 years | 1.0 (ref) |  |  |  |
| 4.01 – 7 years | 0.30 (0.04-2.42) | 0.26 | 0.25 (0.03-2.52) | 0.24 |
| >7 years | 0.02 (0.01-0.20) | 0.001 | 0.03 (0.01-0.33) | 0.003 |

**Supplementary Table S9.** Two factors are associated with bilateral CCL rupture risk reduction among 38 dogs with at least one CCL rupture diagnosis. Dogs with CCL rupture were divided into three categories based upon age at first rupture [less than 4 years (n=13 dogs), 4.01-7 years (n=8 dogs), more than 7 years (n=20 dogs)]. Gonad exposure categories: >24 months = avoidance of gonad removal during the first 24 months of life; <24 months = early endocrine disruption. Adjusted odds ratios (OR) and 95% confidence interval (95% CI) of bilateral rupture were generated using logistic regression. Analysis excludes four dogs with missing values for age at first CCL rupture. Strong risk reduction in the oldest age at first rupture category reflects that no dogs in this study cohort with age at first rupture after 9 years of age developed contralateral CCL rupture.

| **Variable** | **Unadjusted HR (95% CI)** | **p-value** | **Adjusted HR (95% CI)** | **p-value** |
| --- | --- | --- | --- | --- |
| **Parity** | | | | |
| Non-parous | 1.0 (ref) |  |  |  |
| Parous | 0.26 (0.06-1.23) | 0.09 | 0.07 (0.01-0.81) | 0.03 |
| **Gonad Exposure** | | | | |
| >7 years | 1.0 (ref) |  |  |  |
| 4.5 – 7 years | 1.11 (0.22-5.51) | 0.90 | 1.10 (0.18-6.80) | 0.92 |
| 2.0 – 4.5 years | 1.68 (0.40-7.07) | 0.48 | 0.29 (0.03-3.20) | 0.31 |
| **Adult Height** | | | | |
| >61.0 cm | 1.0 (ref) |  |  |  |
| 61.0 cm | 1.25 (0.37-4.27) | 0.72 | 4.30 (0.53-34.72) | 0.17 |
| **Body Condition** | | | | |
| Precedent overweight | 1.0 (ref) |  |  |  |
| Not overweight | 2.18 (0.28-17.06) | 0.46 | 0.68 (0.05-8.72) | 0.77 |

**Supplementary Table S10.** Unadjusted and adjusted hazard ratios (HR) for any cranial cruciate ligament (CCL) rupture generated from Cox proportional hazards model in 26 tall females (adult height > 61.0 cm) that reached reproductive maturity (gonad removal > 24 months). HR (95% CI) = Hazard Ratio and 95% confidence interval. For each risk variable, adjusted HR includes the other three variables in a multivariate analysis. Parity categories: Non-parous: females with no offspring; Parous: females producing offspring. Body Condition categories: Owner-reported body condition was temporally matched with age at CCL rupture so dogs could be categorized as: (1) overweight prior to CCL rupture (i.e., precedent overweight); or (2) not overweight prior to CCL rupture (i.e., not overweight). cm = centimeters; ref = reference group.

| **Variable** | **Dogs Eligible for this Study (n=127)** | **Other Dogs Enrolled in EARS (n=298)** | **p-value**† |
| --- | --- | --- | --- |
| **Residence** | 37 states and Canada | 44 states and Canada |  |
| **Sex** |  |  | 0.66 |
| Females, n (%) | 79 (62.2) | 192 (64.4) |  |
| Males, n (%) | 48 (37.8) | 106 (35.6) |  |
| **Age at Death,** median (range), in years | 14.1 (13.1-16.5) | 13.8 (13.0-16.5) | < 0.001 |
| **Cause of Death** |  |  | 0.46 |
| Sudden Death, n (% total) | 14 (11.0) | 33 (11.1) |  |
| Degenerative Joint Disease/Frailty, n (% total) | 28 (22.0) | 88 (29.7) |  |
| Cancer, n (% total) | 31 (24.4) | 71 (24.0) |  |
| Other, n (% total) | 41 (32.3) | 74 (25.0) |  |
| Insufficient Information, n (% total) | 13 (10.2) | 30 (10.1) |  |
| **Gonad Exposure,** median (range), in years | 5.0 (0.3-16.5) | 5.2 (0.3-16.1) | 0.46 |
| **Gonad Exposure** <**24 months (EED_24m_),** n (%) | 34 (26.8) | 84 (28.2) | 0.81 |
| **Body Condition** |  |  |  |
| Ever Overweight, n (% total) | 24 (18.9) | 24/104 (23.1) | 0.52 |

**Supplementary Table S11.** Characteristics of 127 dogs in the Exceptional Aging in Rottweilers Study (EARS) that were eligible for the current report versus other dogs enrolled in EARS (N=298) that were not eligible for this report. Eligibility for the current report was restricted to only those dogs enrolled in EARS that had extensive data collection during an in-home visit, including standardized orthopedic examination which enabled a standardized assessment of lifetime cranial cruciate ligament (CCL) status. The table shows that the sample of eligible dogs are representative of the overall EARS population on the basis of several relevant characteristics, including widespread geographic residence and duration of lifetime gonad exposure, including the proportion of dogs that experienced early endocrine disruption, i.e., gonad removal during the 24-month developmental period (EED_24m_). †p-values based upon chi-square test statistic for categorical variables, t test for continuous variables.

| **Variable** | **Dogs with Veterinarian-diagnosed Unilateral CCL Rupture** | | | | **p-value**† |
| --- | --- | --- | --- | --- | --- |
|  | **Open Surgery or Laxity-based CCL Rupture Diagnosis (n=15)** | | **Periarticular Fibrosis-based CCL Rupture Diagnosis (n=9)** | |  |
| **Sex,** n (% females) | 9 | (60.0) | 5 | (55.6) | 0.83 |
| **Age at First Rupture,** in years†† | 10.0 | (6.4, 12.0) | 9.9 | (7.2, 10.8) | 0.97 |
| **Gonad Exposure** |  | |  | |  |
| Duration of Gonad Exposure, in years†† | 5.3 | (2.0, 8.3) | 4.0 | (2.2, 6.5) | 0.60 |
| Gonad Removal ≤24 months, n (%) | 4 | (27.0) | 2 | (22.0) | 0.81 |
| **Adult Height** |  | |  | |  |
| Adult Height, in centimeters†† | 63.5 | (61.0, 66.0) | 61.6 | (61.0, 64.1) | 0.20 |
| Sex-Specific – Tall, n (%) | 12 | (80.0) | 6 | (66.7) | 0.47 |
| **Body Condition** |  | |  | |  |
| Precedent Overweight, n (%) | 4 | (26.7) | 1 | (11.1) | 0.36 |

**Supplementary Table S12.** Dogs with veterinarian-diagnosed unilateral cranial cruciate ligament (CCL) rupture: Comparison of dogs with open surgery or laxity based CCL rupture diagnosis (n=15 dogs) versus periarticular fibrosis-based CCL rupture diagnosis (n = 9 dogs). Tall adult height = females > 61.0 cm, males > 64.8 cm. †p-values based upon chi-square test statistic for categorical variables, t test for continuous variables. ††Values for continuous variables are expressed as median (interquartile range).


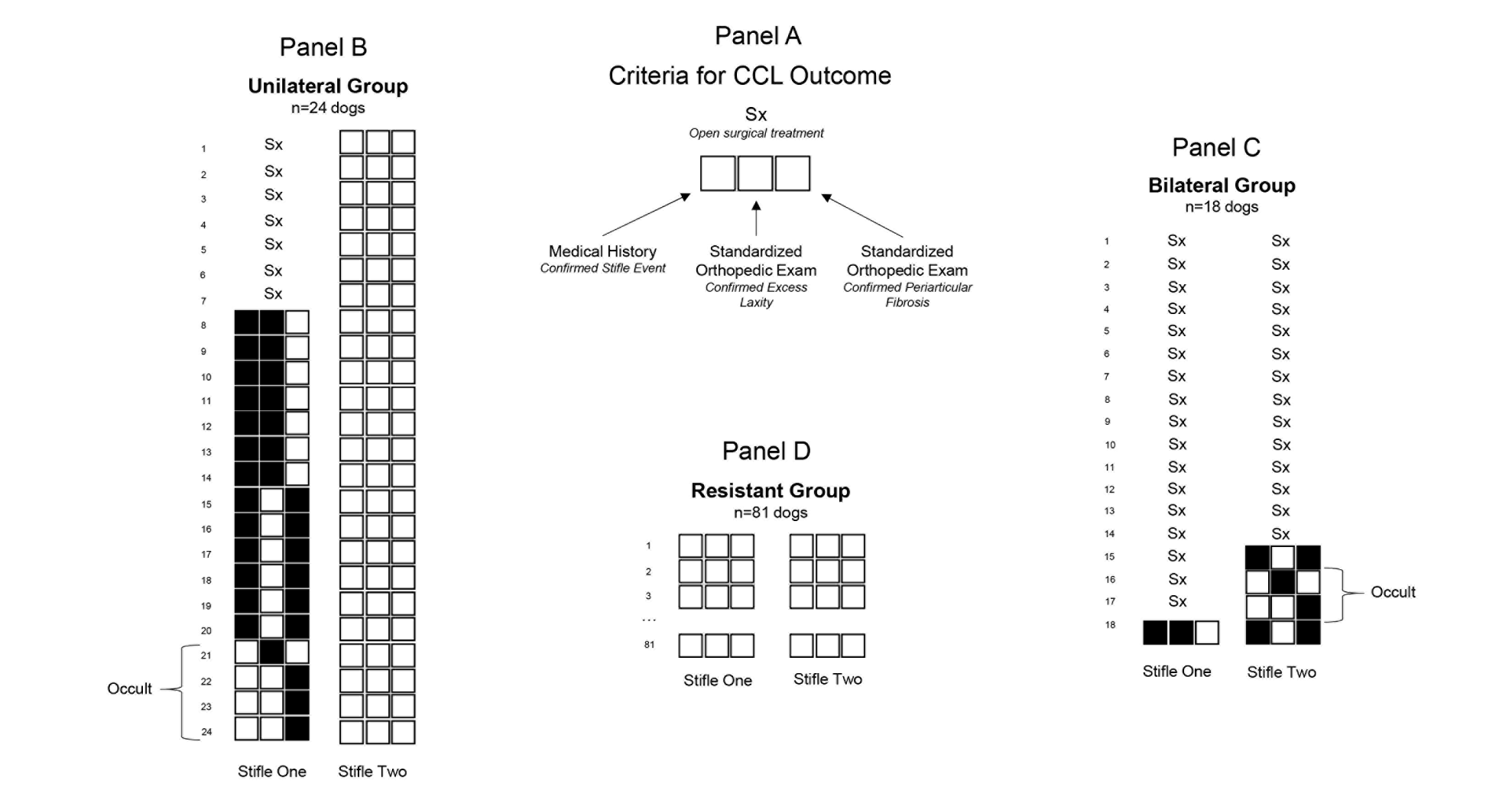


**Supplementary Figure S1**. Criterial basis for lifetime cranial cruciate ligament (CCL) outcome assignment: Open surgery, medical history, physical findings during near-end-of-life standardized orthopedic examination. **Panel A**. Criteria used for assignment of CCL outcome. In some dogs, diagnosis was made at the time of open surgical treatment (designated as “Sx”). For each dog that did not undergo open stifle surgery, three criteria boxes are shown for each stifle. Left box is checked for stifles for which medical history revealed a suspected CCL rupture-associated acute lameness. Middle box is checked for those stifles with laxity-based diagnosis of CCL rupture at time of near-end-of-life standardized orthopedic physical examination. Right box is checked for stifles with periarticular fibrosis-based diagnosis of CCL rupture at time of standardized exam. **Panel B**. Pictorial representation of the diagnostic paths for each stifle in the 24 dogs in the Unilateral Rupture group. **Panel C**. Pictorial representation of the diagnostic paths for each stifle in the 18 dogs in the Bilateral Rupture group. **Panel D**. Pictorial representation of the diagnostic results for the 81 dogs in the Resistant group. All dogs in the Resistant group had no medical history of stifle injury or stifle surgery, and on examination of both stifles showed no cranial-caudal instability during cranial drawer and tibial thrust tests, no thickening of periarticular stifle tissue indicative of chronic CCL rupture. Therefore, for dogs in the Resistant group, each of the three criteria boxes are empty for both stifles, indicating lifelong avoidance of veterinarian-diagnosed CCL rupture. Six dogs with occult CCL rupture – no clinical history of an event suggestive of CCL rupture reported by owner or veterinarian – are identifiable in **Panel B** and **Panel C** as an empty left box. The figure illustrates that excessive cranial-caudal laxity was not the sole criterion used in diagnosis of CCL rupture at time of standardized near-end-of-life examination.











**

**

**Supplementary Figure S2**. Sensitivity analysis to assess the potential impact that CCL rupture underdetection might exert on the associations between gonad exposure, adult height, and CCL rupture found in our primary analysis of dogs in this cohort. Because misclassification of outcome in dogs assigned to the three categories of CCL outcome – RESISTANT, UNILATERAL RUPTURE, or BILATERAL RUPTURE – may lead to spurious associations between risk variables and CCL outcome, a random reassignment sensitivity analysis was conducted to test the robustness of our main findings. False negative results – dogs scored as RESISTANT that actually had UNILATERAL RUPTURE, dogs with UNILATERAL RUPTURE that actually had BILATERAL RUPTURE – are of particular concern to investigators. Therefore, the association between CCL rupture risk and two potential risk reduction phenotypes – short adult height, avoidance of gonad removal during the developmental period – were re-analyzed after random reassignment of CCL outcome (Resistant, Unilateral Rupture, Bilateral Rupture) to mimic plausible levels of underdetection and misclassification in the cohort. Random category-specific outcome reassignment enabled comparison of our original data set (Model 1) with alterations in CCL outcome that simulated different levels of CCL underdetection, i.e., false negative rate. Model 2 mimics a 20% false negative (FN) rate, 5% false positive (FP) rate; Model 3 represents a further increase in FN rate only among dogs in the Unilateral group (40% FN) while leaving 20% FN rate among Resistant group; Model 4 represents an increase in FN rate to 40% for dogs originally categorized in the Resistant and Unilateral groups. In this sensitivity analysis, reassignment is restricted to CCL outcomes; there are no reassignments of risk variables.

**Panel A.** Number of dogs in each of the three CCL outcome categories (Resistant, Unilateral, Bilateral) in original data set (Model 1) and after random reassignment of CCL outcome (Models 2, 3, 4) are shown. CCL outcome category-specific fate of dogs after random reassignment of “movers” can be assessed in **Panel A** by looking across rows from original data set (Model 1) to subsequent outcome reassignments depicted in Models 2, 3, and 4. For example, it can be seen that among the 81 Resistant dogs in original data set, 16 of those dogs after reassignment are now treated as members of the Unilateral Rupture group in Model 2. Further examination of that row shows there are no additional movers from the Resistant group in Model 3, whereas 16 additional movers from Resistant group occupy the Unilateral group in Model 4 to satisfy the conditions of 40% FN rate. Inspection of **Panel A** shows the composition of 27 dogs in the Bilateral group in Model 4 consists of 17 of the 18 original members diagnosed with Bilateral Rupture (one dog moved to Unilateral group to mimic 5% FP rate) and 10 movers who were originally members of the Unilateral group. The proportion of original members of the Bilateral Rupture group categorized as Bilateral Rupture after 40% FN, 5% FP (Model 4) can be calculated (17/27 = .63).

**Panel B**. Impact of random outcome reassignment sensitivity analysis on associations between CCL outcome and two risk reduction phenotypes (short adult height, developmental gonad retention). Odds ratios (OR), 95% CI and p values were generated with both risk variables in a logistic regression model. Risk reduction associated with these phenotypes are considered for the outcome of Unilateral Rupture or Bilateral Rupture separately and displayed for original data set and Models 2, 3, and 4 so degree of attenuation of effect size and statistical significance can be assessed. Notably, for the main finding of this study – gonad retention during the 24-month developmental period is associated with strong risk reduction for bilateral rupture – effect size and statistical significance are not markedly attenuated in these models of simulated CCL rupture underdetection.

**Panel C.** To assist in interpretation of results, the proportion of dogs harboring high-risk phenotypes (tall adult height, EED24 months) is depicted across the resultant models of outcome reassignment. Results shown here can be considered explanatory of CCL outcome misclassification-driven attenuation of risk reduction observed in the primary analysis of the original data set (Model 1) (shown in **Panel B**). For example, dilution of the proportion of dogs in the Unilateral Rupture group with tall adult height from 67% (Model 1) to 50% (Model 4) shown in **Panel C** was associated with attenuation of tall adult height as a significant risk factor for Unilateral Rupture (**Panel B**). In contrast, the sustained high proportion of dogs with tall adult height in the Bilateral Rupture group (78% in Model 1 and 78% in Model 4) in **Panel C** is accompanied by a sustained risk reduction associated with short adult height after simulated misclassification (**Panel B**).

**Panel D**. Proportion of dogs with risk phenotypes among random “movers” are representative of CCL outcome group of origin. To assist in interpretation of results, the proportion of dogs harboring high-risk phenotypes is shown for “movers” and for dogs in the original CCL outcome categories. Histograms on the left provide visual comparison of high-risk phenotype prevalence within the Resistant group by comparing 81 dogs in the original data set (Model 1) versus the 32 former members of the Resistant group that were reassigned (“movers”, **panel A**) to Unilateral group to mimic CCL rupture underdetection in the Resistant group, i.e. dogs categorized as Resistant in our primary analysis that became categorized as Unilateral Rupture after simulated misclassification (Models 2, 3, and 4). Similarly, histograms on the right provide visual comparison of high-risk phenotype prevalence within the Unilateral group by comparing 24 dogs in the original data set (Model 1) versus the 10 former members of the Unilateral group that were reassigned (“movers”, **panel A**) to Bilateral group to mimic CCL rupture underdetection in the Unilateral group, i.e. dogs categorized as Unilateral in our primary analysis that became categorized as Bilateral Rupture after simulated misclassification (Models 2, 3, and 4). Results show that risk profile in random movers from Resistant group and the Unilateral group are strongly representative of their original CCL outcome group.

**

**

**Supplementary Figure S3**. Relationship between age at first cranial cruciate ligament (CCL) rupture and interval to contralateral rupture in 16 dogs with bilateral CCL rupture. There was a trend for earlier age at first rupture to be associated with shortened interval to contralateral ligament failure (r_s_ =.431; Spearman R^2^=.19; p=.10).





**Supplementary Figure S4.** Histogram showing the sexually dimorphic adult height distribution among 76 females and 47 males in the cohort (n = 123) from the Exceptional Aging in Rottweilers Study. Number above bar = number of dogs; cm = centimeters.
